# Estimation and Validation of an Effective Ergothioneine Dose for Improved Sleep Quality Using Physiologically Based Pharmacokinetic Model

**DOI:** 10.1002/fsn3.70382

**Published:** 2025-06-05

**Authors:** Hitoshi Okumura, Yudai Araragi, Kentaro Nishioka, Reiya Yamashita, Toshihide Suzuki, Hiroshi Watanabe, Yukio Kato, Norihito Murayama

**Affiliations:** ^1^ Research Institute Suntory Global Innovation Center Ltd. Kyoto Japan; ^2^ Faculty of Pharmacy Kanazawa University Kanazawa Japan

**Keywords:** brain, curiosity, ergothioneine, physiologically based pharmacokinetic model, sleep quality

## Abstract

A four‐week administration of 20 mg/day ergothioneine (EGT), a strong antioxidant, improves sleep quality; however, its effect at lower doses remains unclear. This study estimated the lower effective doses of EGT using a physiologically based pharmacokinetic (PBPK) model in two clinical trials. In Study 1, participants received 5 or 10 mg/day of EGT for 8 weeks, and their plasma and blood EGT concentrations were measured. An optimized PBPK model incorporating absorption, distribution, and excretion was assembled. Our results showed that 8 mg/day of EGT for 16 weeks was optimal for attaining an effective plasma EGT concentration. In Study 2, a randomized, double‐blind, placebo‐controlled study, participants received 8 mg/day EGT or a placebo for 16 weeks. The subjective sleep quality was significantly improved in the EGT group than in the placebo group (*p* < 0.05). This is the first study to propose a strategy to estimate lower effective doses based on the PBPK model.

## Graphical abstract

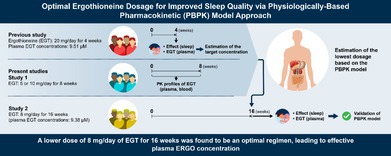

## 1 Introduction

Ergothioneine (EGT) is a sulfur‐containing amino acid that is abundant in mushrooms and has potent antioxidant and anti‐inflammatory properties (Paul and Snyder 2010). In mammals, EGT is not generated in the body but is acquired from the diet via the carnitine/organic cation transporter OCTN1/SLC22A4 (Gründemann et al. 2005; Kato et al. 2010), which is widely expressed in various organs and cells and is responsible for the unique tissue distribution of EGT. OCTN1/SLC22A4 may be expressed in the proximal tubules and is involved in efficient EGT reabsorption (Kato et al. 2010; Tamai et al. 2004); its plasma concentration after oral administration is quite stable and gradually increases after repeated dosing on a multi‐day basis. For example, clinical trials have shown that plasma EGT concentrations increase progressively over 7 days of repeated dosing (Cheah et al. 2016). Furthermore, blood concentrations of EGT increase after Day 8 when EGT intake is interrupted, and they continue to increase until Day 35 (Cheah et al. 2016). The delayed increase in EGT concentration in the blood, compared with that in the plasma, can be interpreted as its efficient uptake by undifferentiated blood cells, which express high levels of OCTN1/SLC22A4 in the bone marrow, and subsequent differentiation to mature blood cells that enter the circulation (Kato et al. 2010; Kobayashi et al. 2004).

EGT has been proposed to possess various health benefits. For example, cross‐sectional studies have confirmed a correlation between plasma EGT levels and the occurrence of dementia, Parkinson's disease, and frailty (Cheah et al. 2016; Hatano et al. 2016; Kameda et al. 2020; Teruya et al. 2021). Longitudinal studies have shown that the risk ratios for cardiac disease and cognitive decline decrease with an increase in plasma EGT (Smith et al. 2020; Wu et al. 2022). Randomized, double‐blind clinical trials have shown that EGT intake can improve sleep quality and cognitive function (Katsube et al. 2022; Watanabe et al. 2020). Katsube et al. (2022) showed that a 4‐week intake of 20 mg EGT resulted in an increased N2 stage and decreased N1 stage during non‐rapid eye movement (NREM) sleep, as well as a reduction in the frequency of waking after sleep onset (WASO), as assessed by electroencephalography. An experimental in vivo animal study showed that EGT ingestion ameliorates stress‐induced sleep disturbances in rats (shortening of sleep onset latency, increased sleep, and improvement in fragmented sleep; Matsuda et al. 2020). However, sleep quality improvement in humans has been demonstrated only at EGT concentrations of 20 mg/day (Katsube et al. 2022), while its efficacy at lower doses remains unknown. The average plasma concentration of EGT at 20 mg for 4 weeks is 9.51 μM (Katsube et al. 2022), implying that improvement in sleep quality can be expected under conditions of administration that reach this plasma concentration.

Physiologically based pharmacokinetic (PBPK) modeling is a mathematical analysis quantitatively describing and predicting the absorption, distribution, metabolism, and excretion of small‐molecule drugs and biologics (Huang et al. 2013). Therefore, the PBPK model can be potentially utilized to estimate the minimum ingestion dose of healthy food active ingredients required for beneficial effects if the effective concentration of the corresponding ingredient in the plasma or organs is available. However, a PBPK model‐based approach for dose optimization has not been generally applied to food ingredients. The absorption and disposition kinetics of EGT have been studied in rodents (Kato et al. 2010; Sugiura et al. 2010), while plasma and blood concentration profiles after repeated oral administration of EGT have been clarified in humans (Cheah et al. 2017), promoting successful construction of the PBPK model. Notably, EGT membrane permeability is limited and observed in the presence of its specific transporter, OCTN1 (Gründemann et al. 2005; Kato et al. 2010). This may imply the nonlinear absorption, distribution, and excretion of EGT owing to saturation of the transporter at higher concentrations, potentially leading to difficulty in model construction.

In this study, we aimed to estimate lower effective doses of EGT for improved sleep quality based on PBPK modeling using two clinical studies. A pharmacokinetic study (Study 1) was initially performed, wherein 5 and 10 mg/day EGT was administered for 8 weeks, and the plasma and blood EGT levels were measured to construct the PBPK model. Study 2 evaluated the effect of EGT on sleep quality by measuring cortical volume, neuronal density in the cerebral medulla, and curiosity as exploratory assessments under optimized EGT dose conditions. Curiosity was measured by considering the previous report demonstrating improved novel object recognition in mice following EGT administration (Nakamichi et al. 2021). Study 2 also examined plasma EGT concentrations to verify the accuracy of the PBPK model created in Study 1.

## 2 Materials and Methods

### 2.1 Study 1 Design

#### 2.1.1 Study Design and Population

A randomized, double‐blind clinical trial was designed and conducted over an 8‐week period to evaluate plasma and blood concentration trends following EGT ingestion (5 and 10 mg/day; GMP‐certified > 99.5% L‐EGT, Tetrahedron, Rotterdam, the Netherlands). Participants were instructed to take the capsules in the morning. If they could not take them in the morning, they were allowed to take them in the afternoon after recording their use in their daily logbooks. Blood samples were collected at baseline and after 8, 14, 21, 28, and 56 days of EGT intake. On the day of blood collection, the participants fasted for at least 8 h. EGT was administered following blood collection. The sample size was determined based on the pharmacokinetic study previously reported for the sleeping drug diazepam (Watanabe et al. 1990), but was larger than that, as the variability in plasma EGT concentrations in the previous study (Katsube et al. 2022) was greater than the variability in the study of diazepam (Watanabe et al. 1990).

Participants were selected from 34 Japanese volunteers recruited for the study based on the following criteria: individuals who (1) were healthy men and women, aged 30–59 years at the time of consent acquisition, and employees of Suntory Holdings Limited; and (2) demonstrated a comprehensive understanding of the study's objective and procedures and provided written consent to participate. The exclusion criteria were as follows: (1) history of major surgery on the gastrointestinal tract; (2) regular consumption of mushrooms (excluding maitake and kikurage), legumes, or oat bran at least twice a week; (3) presence of diabetes, heart, liver, or kidney disease; (4) a history of cardiovascular disease; (5) habitual consumption of > 60 g of alcohol per day on average; (6) concurrent participation or intention to participate in other clinical studies; and (7) deemed unsuitable for participation by the study investigator.

#### 2.1.2 Randomization and Blinding

Overall, 28 eligible participants were randomized into two equal‐sized groups of 14 individuals, using minimization methods, with age, sex, body mass index (BMI), and pre‐intervention plasma EGT concentration as allocation factors. The investigators and participants were double‐blinded to treatment regimens. Eligible study participants were randomized to either Study Product 1 (containing 5 mg EGT) or Study Product 2 (containing 10 mg EGT), administered as one capsule per day for 8 weeks. A third‐party allocation agency managed allocation information and ensured that double‐blindness was maintained until the data were analyzed.

#### 2.1.3 Construction of the PBPK Model

The PBPK model and physiological parameters are shown in Figure S1 and Table 1, respectively. Mass‐balance equations are provided in the Supporting Information S1. The fecal excretion of EGT was assumed to be negligible to minimize the number of optimized parameters, as gastrointestinal absorption of EGT after oral administration of [^3^H]EGT was almost complete in mice (Sugiura et al. 2010). The liver was conceptualized as comprising five tandem compartments, reflecting the rapid hepatic uptake kinetics of [^3^H]EGT under blood‐flow‐limited conditions (Sugiura et al. 2010; Figure S1). This modeling approach aligns with a previous report on the PBPK models designed for drugs showing highly efficient hepatic uptake (Watanabe et al. 2009). The blood concentrations of [^3^H]EGT following oral administration exhibited a much longer lag time than plasma in mice, suggesting that EGT is taken up by the red blood cell (RBC) precursor (Kato et al. 2010). Therefore, an RBC precursor compartment was assumed to be present (Figure S1). Large organs expressing OCTN1 (including muscle, adipose tissue, and skin) were also included in the PBPK model. The uptake of EGT in these organs and the liver was assumed to be saturable via OCTN1‐mediated transport, as described by the Michaelis–Menten equation. Therefore, these organs were assumed to consist of extracellular and intracellular compartments (Figure S1). EGT is reabsorbed in the kidney by OCTN1 following glomerular filtration (Kato et al. 2010); therefore, it was assumed to be transferred to the proximal duct compartment by the glomerular filtration rate and subsequently reabsorbed by the saturable process in the kidney (Figure S1).

**TABLE 1:** Parameters in the PBPK model.^a^

	Values±SD	Units	Note and references
Daily ingestion rate of EGT via diet, *R*	1.36 ± 0.58	μmol/h	Optimized
Absorption rate constant, *k*_a_ ^b^	6.00	/h	Fixed
**Volumes of organs**			
Plasma, *V*_p_	3.00	L	Fixed (Davies and Morris 1993)
Blood	5.20	L	Fixed (Davies and Morris 1993)
RBC, *V*_RBC_ ^c^	2.29	L	Fixed (Davies and Morris 1993)
Adipose (intracellular space), *V*_a_ ^c^	10.0	L	Fixed (Davies and Morris 1993)
Adipose (extracellular space), *V*_ae_ ^c^	2.00	L	Fixed (Davies and Morris 1993)
Liver (intracellular space), *V*_h_ ^c^	1.69	L	Fixed (Davies and Morris 1993)
Liver (extracellular space), *V*_he_ ^c^	0.338	L	Fixed (Davies and Morris 1993)
Skin (intracellular space), *V*_s_ ^c^	7.8	L	Fixed (Davies and Morris 1993)
Skin (extracellular space), *V*_se_ ^c^	1.56	L	Fixed (Davies and Morris 1993)
Muscle (intracellular space), *V*_m_ ^c^	35.0	L	Fixed (Davies and Morris 1993)
Muscle (extracellular space), *V*_me_ ^c^	7.00	L	Fixed (Davies and Morris 1993)
Renal tubular duct, *V*_d_	0.127	L	Fixed (Sarashina et al. 2020)
**Plasma flow rate** ^**c**^			
Adipose, *Q*_a_	8.74	L/h	Fixed (Davies and Morris 1993)
Liver, *Q*_h_	48.7	L/h	Fixed (Davies and Morris 1993)
Skin, *Q*_s_	18.0	L/h	Fixed (Davies and Morris 1993)
Muscle, *Q*_m_	45.0	L/h	Fixed (Davies and Morris 1993)
Urinary flow rate, *Q*_u_	0.0583	L/h	Fixed (Davies and Morris 1993)
Glomerular filtration rate	7.50	L/h	Fixed (Davies and Morris 1993)
**RBC parameters**			
RBC differentiation, *k*_1_ ^d^	0.0104	/h	Fixed (Yasukouchi et al. 1999)
RBC degradation, *k*_b_ ^e^	0.000963	/h	Fixed (Obara 1962)
**Maximum uptake rate** ^**f**^			
Liver, *V*_maxH_	10,227	μmol/h	Fixed
Duct, *V*_maxD_	121 ± 71	μmol/h	Optimized
RBC precursor, *V*_maxP_	11.1 ± 0.9	μmol/h	Optimized
Michaelis constant, *K*_m_ ^g^	21.0	μM	Fixed (Gründemann et al. 2005)
**Tissue‐to‐plasma concentration ratio** ^**h**^			
Adipose, *K*_pA_	17.5		Fixed
Liver, *K*_pH_	80.1		Fixed
Skin, *K*_pS_	20.8		Fixed
Muscle, *K*_pM_	5.06		Fixed
**Initial values for fitting**			
5 mg/day			
*C*_Plasma_ ^i^	3.42	μM	Fixed
*C*_RBC_ ^i^	624	μM	Fixed
10 mg/day			
*C*_Plasma_ ^i^	3.51	μM	Fixed
*C*_RBC_ ^i^	562	μM	Fixed
20 mg/day			
*C*_Plasma_ ^i^	2.97	μM	Fixed
*C*_RBC_ ^j^	594	μM	Fixed
**Initial values for simulation**			
8 mg/day			
*C*_Plasma_ ^j^	3.16	μM	
*C*_RBC_ ^k^	594	μM	

Abbreviations: EGT, ergothioneine; *K*_m_, Michaelis constant; PBPK, physiologically based pharmacokinetic; RBC, red blood cell; SD, standard deviation; *V*_max_, maximum uptake rate.

^a^ Each value represents per human value assuming 70 kg body weight.

^b^ Fixed as gastric emptying rate (Kanamitsu et al. 2000) assuming rapid EGT absorption.

^c^ Cited from Davies and Morris (1993), where the volume of RBC and plasma flow rate were calculated using the hematocrit value, and the volume of extracellular space was assumed to be 0.2 times that of each tissue.

^d^ Cited from Yasukouchi et al. (1999), where the half‐life of RBC differentiation was assumed to be 2.75 days.

^e^ Cited from the study conducted by Obara (1962), where the half‐life of RBC was assumed to be 30 days.

^f^ *V*_max_ values in each tissue were fixed to be proportional to the OCTN1 mRNA level (see text for details). In the liver, *V*_maxH_/*K*_m_ was set to be 10 times (*Q*_h_), assuming blood‐flow limited uptake.

^g^ Fixed to the Km value for EGT uptake by human OCTN1.

^h^ Assumed to be the same values as those observed in mice (Araragi et al., manuscript in preparation).

^i^ The blank value obtained in each clinical study.

^j^ The average of blank values at 5 mg/day and 10 mg/day.

^k^ The average of blank values at 5 mg/day, 10 mg/day, and 20 mg/day.

OCTN1 mRNA expression levels in the liver, muscle, adipose tissue, and skin were obtained from three databases, including HPA RNA‐seq normal tissues, RNA sequencing of total RNA from 20 human tissues, and the Illumina bodyMap2 transcriptome (https://www.ncbi.nlm.nih.gov/gene/6583). Subsequently, the *V*_max_ values (per gram of tissue) in the liver, muscle, adipose, and skin were assumed to be proportional to the average of the three values (0.147, 0.464, 0.209, and 0.131), whereas the Michaelis constant (*K*_m_) value was fixed to the reported value for EGT uptake by human OCTN1 (21 μM; Gründemann et al. 2005). Furthermore, the ratio of *V*_max_/*K*_m_ to intrinsic efflux clearance (PS_eff_) in each organ was assumed to mirror the tissue‐to‐plasma concentration ratio of EGT obtained in mice (80.1, 5.06, 17.5, and 20.8 in the liver, muscle, adipose, and skin, respectively, assuming steady‐state and linear conditions; Araragi et al., manuscript in preparation).

EGT concentrations in RBCs (*C*_RBC_) were calculated from the blood and plasma concentrations (*C*_Blood_ and *C*_Plasma_, respectively) measured in clinical studies based on the following equation:

(1) $$C_{\mathrm{RBC}}=\frac{C_{\text{Blood}}+C_{\text{Plasma}}\times \left(1-H_{\mathrm{ct}}\right)}{H_{\mathrm{ct}}}$$

where Hct refers to hematocrit. Fitting of the PBPK model was performed to the average values of *C*_Plasma_ and *C*_RBC_ values to optimize three parameters, such as daily ingestion rate of EGT via diet (*R*), maximum rate in RBC precursor (*V*_maxP_), and maximum rate in renal reabsorption (*V*_maxD_) using a nonlinear least‐squares fitting computer program Napp (version 3.071β; https://www.p.chiba‐u.jp/lab/cpp/napp.html). In the fitting, the initial value for *C*_Plasma_ at 5, 10, and 20 mg/day was set as the blank value for each dose, whereas that for *C*_RBC_ at 5 and 10 mg/day was obtained based on Equation (1) using blank values for *C*_Plasma_ and *C*_Blood_ at each dose. The initial value for *C*_RBC_ at 20 mg/day was set as the average of the blank values observed at 5 and 10 mg/day. In the simulation, the initial value for *C*_Plasma_ was set as the average of the blank values obtained at 5, 10, and 20 mg/day, whereas that for *C*_RBC_ was set as the average of the blank values obtained at 5 and 10 mg/day. The initial values in the other organs were numerically calculated based on mass‐balance equations, assuming a steady state (see Supporting Information S1).

#### 2.1.4 Sensitivity Analysis

One of the four parameters (*R*, *V*_maxP_, *V*_maxH_, and *V*_maxD_) was changed by 2‐ and 0.5‐fold, and the simulation was performed based on the PBPK model. The sensitivity was obtained as follows:

(2) $$\text{Sensitivity}=\frac{C_{\text{Plasma}\;X-\text{fold}}}{C_{\text{Plasma}}}-1$$

where *C*_Plasma_ represents the simulated plasma EGT concentration at 16 weeks after 8 mg/day daily ingestion, whereas *C*_Plasma X‐fold_ represents the simulated concentration using one of the four parameters that changed 2‐ or 0.5‐fold.

#### 2.1.5 Statistical Analysis

Baseline characteristics *p*‐values for individuals were calculated using Fisher's exact test for sex and independent two‐sample *t*‐tests for all others. Statistical analyses were performed using Microsoft Excel (Microsoft Corp., Redmond, WA, USA), JMP (SAS Institute, Cary, NC, USA), and SPSS Statistics (IBM Corp., Armonk, NY, USA).

### 2.2 Study 2 Design

#### 2.2.1 Study Design and Population

This randomized, double‐blind, placebo‐controlled clinical trial was designed and conducted over 16 weeks to evaluate the effects of 8 mg EGT on sleep quality improvement, compared with that of a placebo. The test capsules consumed by the EGT group comprised 8 mg EGT (GMP‐certified > 99.5% L‐EGT; Tetrahedron), food‐grade dextrin, and calcium stearate, whereas that of the placebo group did not contain EGT. Those capsules could not be distinguished by appearance or smell. It was not specified when the test capsules would be ingested during the day. Blood samples were collected at baseline and after 16 weeks of intake (Week 16). The sample size was designed based on the number of participants in the previous study (Katsube et al. 2022) and was set at the maximum feasible number in this exploratory study.

Participants were selected from 179 Japanese volunteers recruited for the study. The following individuals were included: (1) healthy men and women aged 40–64 years at the time of consent; (2) those who were aware of memory or attention decline; and (3) those who completely understood the purpose and content of the study and provided written consent to participate. The exclusion criteria were as follows: (1) treatment for sleep disorders or were determined to have them by the study investigator; (2) presence of sleep apnea‐hypopnea, nocturia (twice or more); (3) working night shifts; (4) planned changes in their sleeping conditions; (5) consumption of > 20 g of alcohol per day on average; (6) regular ingestion of supplements that may affect their sleep conditions and cognitive function; (7) regular consumption of > 100 g of mushrooms; (8) regular consumption of medicines; (9) undergoing treatment or medications for dementia, psychiatric disorder, cerebrovascular disease, cancer, or tuberculosis; (10) presence of diabetes or respiratory, heart, liver, or kidney disease; (11) a history of neurological or psychiatric disease, cardiac insufficiency, circulatory system disease, cancer, or tuberculosis; (12) participation or possible participation in other clinical studies; and (13) deemed unsuitable for participation by the study investigator. Eligible participants were further selected by neurocognitive index (NCI) on the Cognitrax (relatively low), state anxiety on the State–Trait Anxiety Inventory (STAI) (relatively high), and total score on the Japanese version of the Pittsburgh Sleep Quality Index (PSQI‐J) (relatively high).

Cognitrax is a computer‐based battery of cognitive function tests that was developed as a Japanese version of CNS Vital Signs (Gualtieri and Johnson 2006). STAI and PSQI‐J are validated Japanese versions of the questionnaire (Doi et al. 2000; Nakazato and Mizuguchi 1982).

#### 2.2.2 Randomization and Blinding

Overall, 100 eligible participants were randomized into two groups, with 50 participants allocated to each group. The randomization process employed sorting blocks based on age, sex, STAI, Cognitrax (NCI), and BMI as allocation factors. The investigators and participants were double‐blinded to treatment regimens. Eligible study participants were randomized to consume either the Study Product (containing 8 mg of EGT) or one capsule of a placebo product per day for 16 weeks. A third‐party allocation agency managed allocation information and ensured that double‐blindness was maintained until the data were analyzed.

#### 2.2.3 Oguri‐Shirakawa‐Azumi Sleep Inventory MA (OSA‐MA) Version

The sleep profiles of the participants were confirmed using the OSA‐MA (Yamamoto et al. 1999). This was converted into a five‐factor response scale (Zc) for Factor I (Sleepiness on rising), Factor II (Initiation and maintenance of sleep), Factor III (Frequent dreaming), Factor IV (Refreshing), and Factor V (Sleep length). The statistical improvement for each factor was examined. Statistical analysis was conducted for each of them since Factor II can be divided into “initiation of sleep” and “maintenance of sleep.” The OSA‐MA questionnaire (Japanese) is attached as Supporting Information S1.

The questionnaire was administered at baseline and at Week 16. Individual scores were calculated based on the 3‐day averages at both time points. Missing values were identified for data not filled in during the morning, and participants with > 2 days of missing data at either baseline or Week 16 were excluded from the analysis.

#### 2.2.4 Measuring the Brain Healthcare Quotient

Brain structure changes were quantitatively assessed by measuring the gray matter (GM)‐Brain Healthcare Quotient (BHQ), which represents the volume of GM, and fractional anisotropy (FA)‐BHQ, which represents the neural density of the white matter, using a previously reported method (Fujino et al. 2022). Briefly, three‐dimensional (3D) T1‐weighted images and diffusion tensor images were obtained while the participants reclined in the magnetic resonance imaging (MRI) scanner and were instructed to rest without engaging in any specific cognitive tasks or thoughts. Especially, 3D T1‐weighted images were used to calculate the GM‐BHQ, and diffusion tensor images were used to calculate the FA‐BHQ. The MRI scans for this study were obtained at the Tokyo Institute of Technology at baseline (July 27, 2021, to August 11, 2021) and Week 16 (November 11, 2021, to December 1, 2021) prior to study initiation.

#### 2.2.5 Other Measurements

Multiple curiosities were measured using three curiosity scales. The first was the Japanese version of the Curiosity and Exploration Inventory, consisting of two factors: “stretching” and “embracing,” as well as the Curiosity and Exploration Inventory‐II (CEI‐II) as a total score (Nishikawa et al. 2015). The second was the Epistemic Curiosity Scale, consisting of diverse and specific curiosity (Nishikawa and Amemiya 2015, 2018). The third was the Interpersonal Curiosity Scale, consisting of three factors: personal emotions, privacy, and attributes (Nishikawa et al. 2022).

#### 2.2.6 Statistical Analysis

The validity of the study was evaluated in 99 participants, excluding one who dropped out owing to relocation. We performed a paired *t*‐test for within‐group comparisons and an independent two‐sample *t*‐test for between‐group comparisons of the changes from baseline to Week 16. Baseline characteristics *p*‐values for individuals were calculated using Fisher's exact test for sex and *t*‐tests for all others. The incidence of adverse events and adverse reactions was evaluated in all 100 study participants after allocation. The number of adverse events, number of occurrences, and incidence rate (number of occurrences/number of participants), as well as the number of adverse drug reactions, number of occurrences, and incidence rate (number of occurrences/number of participants), were tabulated for each group. For incidence rates, Fisher's exact test was used to compare the values between the groups, and *p*‐values were calculated. The intake rate of the test food was calculated for each individual and classified into two categories of intake rate, > 90%/< 90%, and frequency tabulations were performed by category for Full Analysis Set and Per‐Protocol Set. SAS software for Windows (version 9.4; SAS Institute) was used for the analysis, and the statistical significance level was set at *p* < 0.05.

### 2.3 Measurement of Plasma and Blood EGT Concentrations

Blood was collected in collection tubes containing sodium heparin as an anticoagulant. Plasma samples containing EGT were centrifuged (1200 × *g*, 10 min, room temperature or 4°C) to facilitate the separation of plasma from blood. Commercially available isotope‐labeled EGT‐d9 (Toronto Research Chemicals, Toronto, Ontario, Canada) was used as the internal standard (IS). For plasma EGT samples, 600 μL of acetonitrile was added to a mixture containing 30 μL of plasma, 30 μL of extra pure water, and 10 μL of a 10‐μM IS solution. Then, the mixture was vortexed and centrifuged at 10,000 × *g*, 4°C for 5 min. Subsequently, 100 μL of the supernatant was collected and diluted with 100 μL of mobile phase A (details below). For blood EGT samples, 600 μL of acetonitrile was added to a mixture containing 30 μL of blood, 30 μL of extra pure water, and 10 μL of a 100 μM IS solution. The mixture was then vortexed and centrifuged at 10,000 × *g*, 4°C for 5 min. Next, 20 μL of the supernatant was collected and diluted with 80 μL of acetonitrile and 100 μL of mobile phase A (details below). The samples were injected with 2 μL for plasma samples or 1 μL for blood samples and analyzed using an ultra‐high‐performance liquid chromatography–tandem mass spectrometry LC‐20 ad System (Shimazu, Kyoto, Japan) coupled to a QTRAP5500 or API4000 (AB Sciex, Tokyo, Japan) quadrupole tandem mass spectrometry instrument.

Chromatographic separation of EGT was performed using a ZIC‐cHILIC (150 × 2.1 mm, 3 μm; 100 Å, Merck Millipore Corporation, Burlington, MA, USA) column with aqueous 0.1% formic acid as mobile phase A and acetonitrile containing 0.1% formic acid as mobile phase B. Gradient elution was performed as follows: 0–0.5 min, 5% A/95% B; 0.5–10 min, 5% A/95% B to 80% A/20% B; 10–11 min, 80% A/20% B; 11–11.1 min, 80% A/20% B to 5% A/95% B; 11.1–15 min, 5% A/95% B at a flow rate of 0.4 mL/min. The mass spectrometer was operated in multiple reaction monitoring modes with positive electrospray ionization EGT and EGT‐d9 mass transitions of *m/z* 230.0 > 86.4 and *m/z* 239.0 > 195.0, respectively. The curtain gas, collision gas, temperature, Gas1, Gas2, and Declustering potential were set at 30 psi, 5 psi, 400°C, 30 psi, 30 psi, and 50 V, respectively.

The concentration of the samples was determined by weighted (1/*x* ^2^) least square linear regression on the peak area ratio (EGT/IS) using a calibration curve. For plasma samples, the calibration curve included concentrations of 0.1 μM, 0.2 μM, 0.5 μM, 1 μM, 2 μM, 5 μM, and 10 μM, while for blood samples, it included concentrations of 3 μM, 6 μM, 15 μM, 30 μM, 60 μM, 150 μM, and 300 μM. Samples with concentrations exceeding the maximum standard concentration were diluted and remeasured.

The plasma and blood estimations were validated for the calibration curve, accuracy, and precision (intra‐assay and inter‐assay reproducibility), matrix effect, additive recovery, carry‐over, dilution integrity (10‐fold dilution), post‐preparative stability in an autosampler (48 h, 10°C), freeze and thaw stability (three times), short‐term stability at room temperature (24 h) and long‐term stability in a deep freezer (93 days; Tables S1 and S2).

### 2.4 Ethics Statement

This study was conducted in accordance with the guidelines of the Declaration of Helsinki (revised by the Fortaleza Assembly of the World Medical Association). All procedures involving human participants were approved by the following Institutional Review Boards (IRBs): Study 1: Miura Clinic, Medical Corporation Kanonkai IRB (Osaka, Japan, approved on September 24, 2020; approval number: R2009‐1) and Study 2: Institutional Review Board of Kanazawabunko Hospital (Kanagawa, Japan, approved on June 2, 2021). Written informed consent was obtained from all participants prior to their enrollment. Personal information was used in accordance with applicable laws and regulations. This study was registered with the University Hospital Medical Information Network Clinical Trials Registry (UMIN‐CTR) (www.umin.ac.jp/ctr/index.htm; Study 1: September 28, 2020, registration number: UMIN000041910; Study 2: June 17, 2021, registration number: UMIN000044580).

## 3 Results and Discussion

### 3.1 Results of Study 1

#### 3.1.1 Demographic Information and Compliance Assessment

**FIGURE 1:**
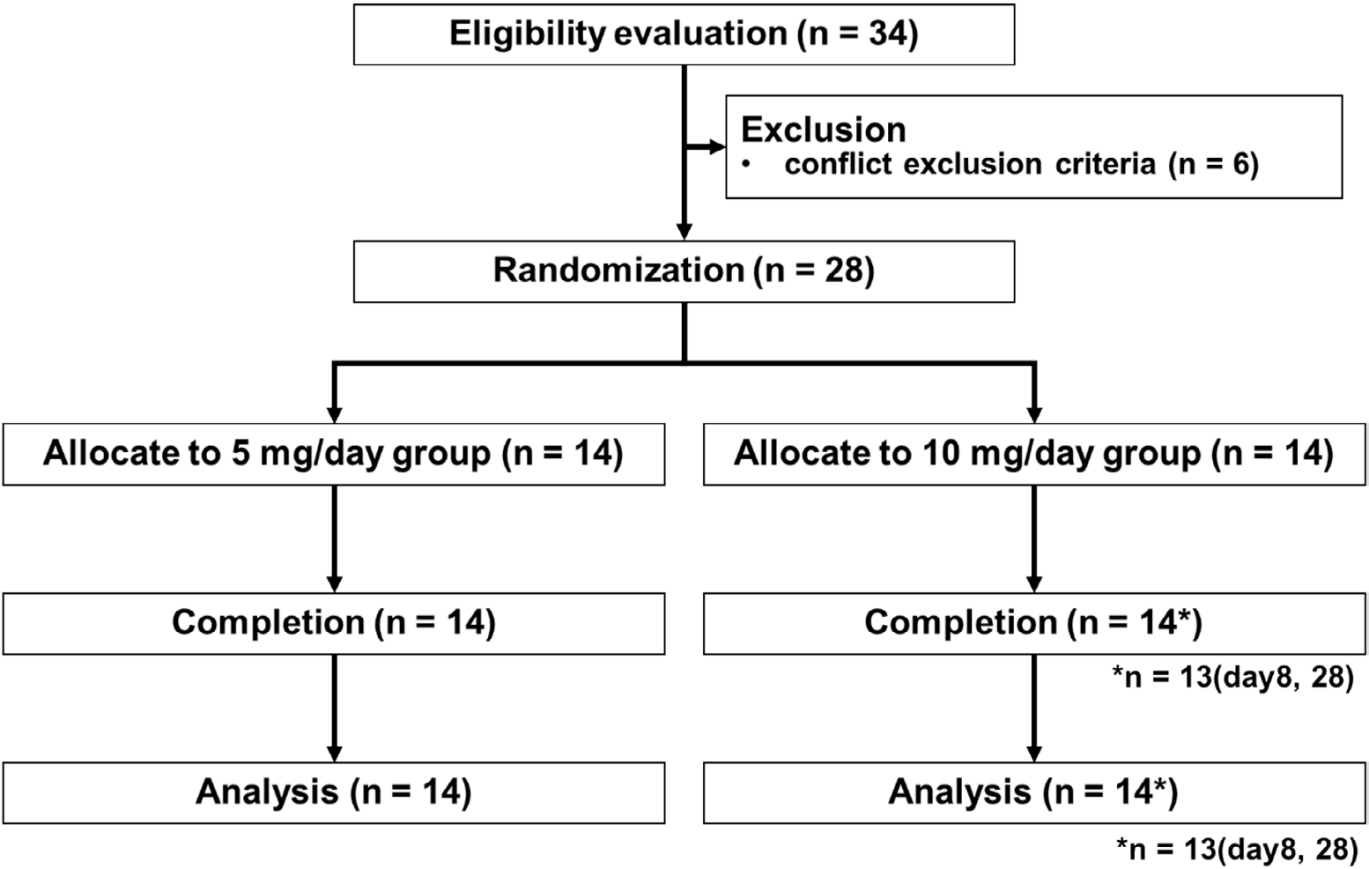
Flow diagram showing participant recruitment for study 1. Although all participants (*n* = 28) completed the study, one participant from the 10 mg/day group was unable to visit on days 8 and 28, and therefore, these data points were excluded.

Overall, 28 participants (14 each in the 5 and 10 mg/day groups) were enrolled in this clinical trial. No participants withdrew by the end of the study; however, data for one participant in the 10 mg/day group were missing on Days 8 and 28 (Figure 1). Of the participants, eight were female (four in the 5 mg/day group and four in the 10 mg/day group), and 20 were male (10 in the 5 mg/day group and 10 in the 10 mg/day group); the mean age, height, weight, BMI, systolic blood pressure, diastolic blood pressure, and heart rate were not significantly different between the groups (Table S3). There were no significant differences in adverse events between the groups in terms of the number of cases, occurrences, and incidence rates. There were no safety issues during the study period. Furthermore, the intake rate of the study tablets was above 99%.

#### 3.1.2 EGT Concentrations in Plasma and Whole Blood

**FIGURE 2:**
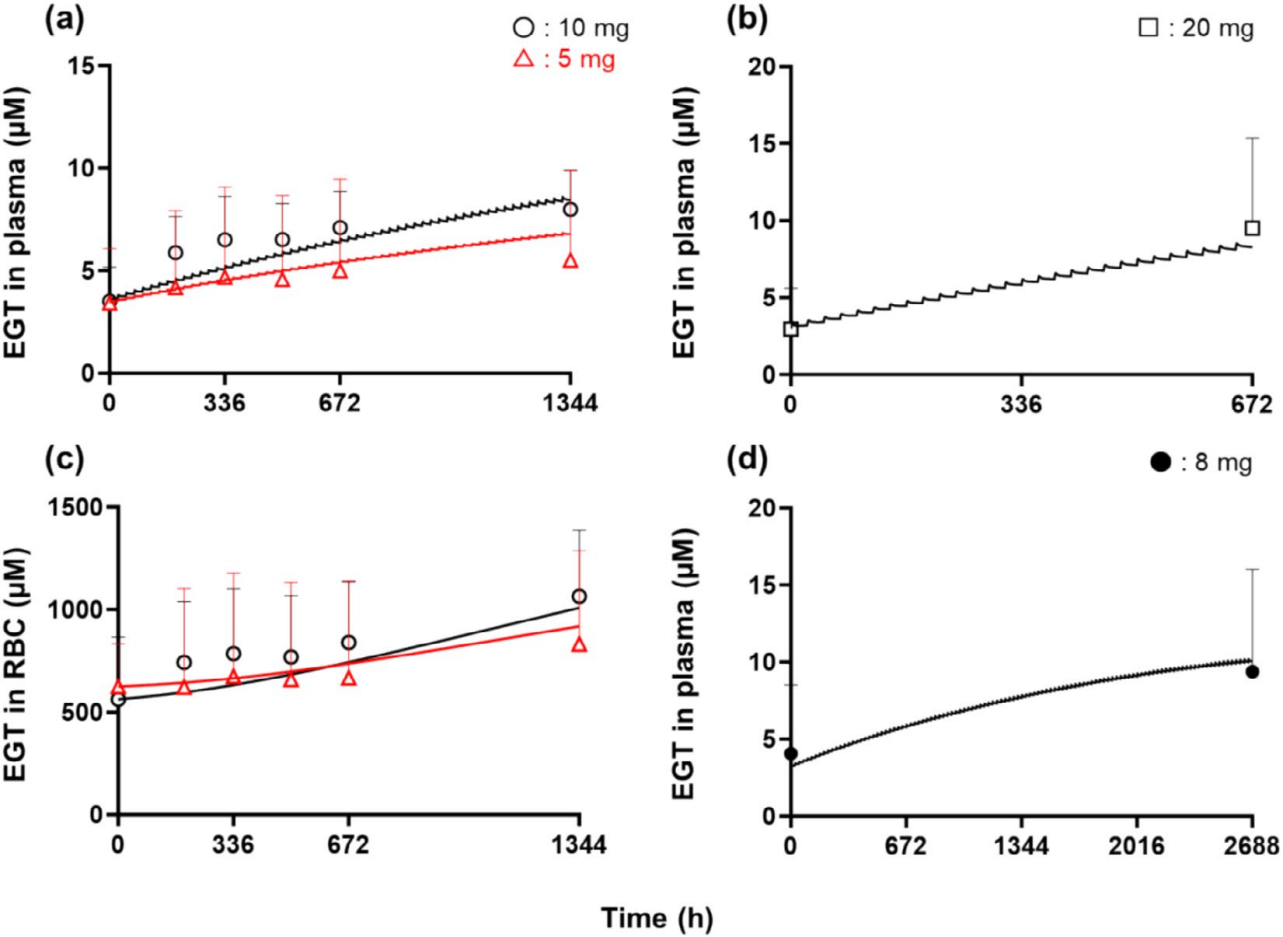
EGT concentration profile in plasma and RBC after repeated oral administration. (a) Open triangles and circles represent plasma EGT concentrations obtained in Study 1 (5 and 10 mg/day, respectively). The solid lines are fitted curves. (b) Squares represent plasma concentrations obtained in the previous clinical study (20 mg/day). (c) Open triangles and circles represent EGT concentrations in RBC obtained in Study 1 (5 mg/day and 10 mg/day, respectively). The solid lines are fitted curves. (d) Closed circles represent plasma concentrations obtained in Study 2 (from 4.07 to 9.38 μM, 8 mg/day). The solid line is a simulation curve. The values are presented as means ± standard deviations (*n* = 14–50). EGT, ergothioneine; RBC, red blood cell.

The plasma EGT concentrations were 3.42 ± 1.74 μM at baseline and 5.52 ± 1.97 μM on Day 56 in the 5 mg/day group. In comparison, they were 3.51 ± 2.75 μM at baseline and 7.99 ± 4.53 μM on day 56 in the 10 mg/day group (Figure 2a). There were no significant differences between the groups in the plasma or blood EGT concentrations at baseline (Table S3). Whole‐blood EGT concentrations were 286 ± 126 μM at baseline and 383 ± 129 μM on Day 56 in the 5 mg/day group, while they were 266 ± 105 μM at baseline and 498 ± 196 μM on Day 56 in the 10 mg/day group (Figure 2c).

#### 3.1.3 Constructing the PBPK Model

Initially, the PBPK model was fitted to the plasma and RBC concentration profiles of EGT at 5, 10, and 20 mg/day (Figure 2a–c), where the data at 5 and 10 mg/day were obtained in Study 1, whereas those at 20 mg/day were previously obtained (Katsube et al. 2022), yielding three optimized parameters (*R*, *V*_maxD_, and *V*_maxP_; Table 1). The fitted lines were almost superimposed on the data (Figure 2a–c), and the coefficient of variation (CV) values of the optimized parameters were within 8%–59%.

#### 3.1.4 Simulating Plasma EGT Profiles at Various Doses to Estimate an Appropriate Dose in Study 2

The plasma EGT concentration profiles at various doses were simulated using the constructed PBPK model to estimate the appropriate EGT dose in Study 2. Plasma EGT concentration simulated during repeated oral administration of 6, 7, 8, and 9 mg/day for 16 weeks yielded 9.16, 9.61, 10.0, and 10.4 μM at 16 weeks using the optimized parameters and setting initial values for *C*_Plasma_ at 3.16 μM (the average of the blank values at 5, 10, and 20 mg/day; Figure 2d, Table 2). As the background values of plasma EGT concentration ranged from 2.97 to 3.51 μM (Figure 2a–c), simulation was also performed when the initial value for *C*_Plasma_ was set at these two values (Table 2).

**TABLE 2:** Simulation of plasma concentration during EGT ingestion at 6, 7, 8, and 9 mg/day.^a^

	Dose (mg/day)	Time after the start of EGT ingestion (week)			
		4	8	12	16
Initial values: *C*_Plasma_: 2.97 μM^b^ *C*_RBC_: 594 μM^c^	6	5.24	6.88	8.15	9.11
	7	5.44	7.23	8.58	9.56
	8	5.65	7.59	9.02	9.99
	9	5.86	7.94	9.43	10.4
Initial values: *C*_Plasma_: 3.16 μM^d^ *C*_RBC_: 594 μM^c^	6	5.37	6.98	8.23	9.16
	7	5.57	7.33	8.66	9.61
	8	5.78	7.69	9.08	10.0
	9	5.99	8.04	9.49	10.4
Initial values: *C*_Plasma_: 3.51 μM^e^ *C*_RBC_: 594 μM^c^	6	5.60	7.15	8.36	9.26
	7	5.81	7.50	8.78	9.70
	8	6.02	7.86	9.21	10.1
	9	6.23	8.21	9.61	10.5

^a^ The simulated plasma EGT concentration was shown as μM.

^b^ The blank value at 20 mg/day.

^c^ The average of the blank values obtained at 5 mg/day and 10 mg/day.

^d^ The average of the blank values obtained at 5 mg/day, 10 mg/day, and 20 mg/day.

^e^ The blank value at 10 mg/day. EGT, ergothioneine.

Katsube et al. (2022) showed that EGT intake at 20 mg/day for 4 weeks maintained the deep stages of non‐REM sleep and reduced mid‐onset arousal. In this study, the average plasma EGT concentration at 4 weeks was 9.51 μM, and this value was used as the target concentration in Study 2. The simulation showed that this concentration was achieved 16 weeks after the start of ingestion at doses of ≥ 7 mg/day (Table 2). However, one of the predicted values at 7 mg/day closely approached the target concentration at 16 weeks (9.56 μM compared to the target of 9.51 μM; Table 2). Therefore, the dose used in Study 2 was 8 mg/day to ensure the beneficial effects of EGT.

### 3.2 Results of Study 2

#### 3.2.1 Demographic Information and Compliance Assessment

**FIGURE 3:**
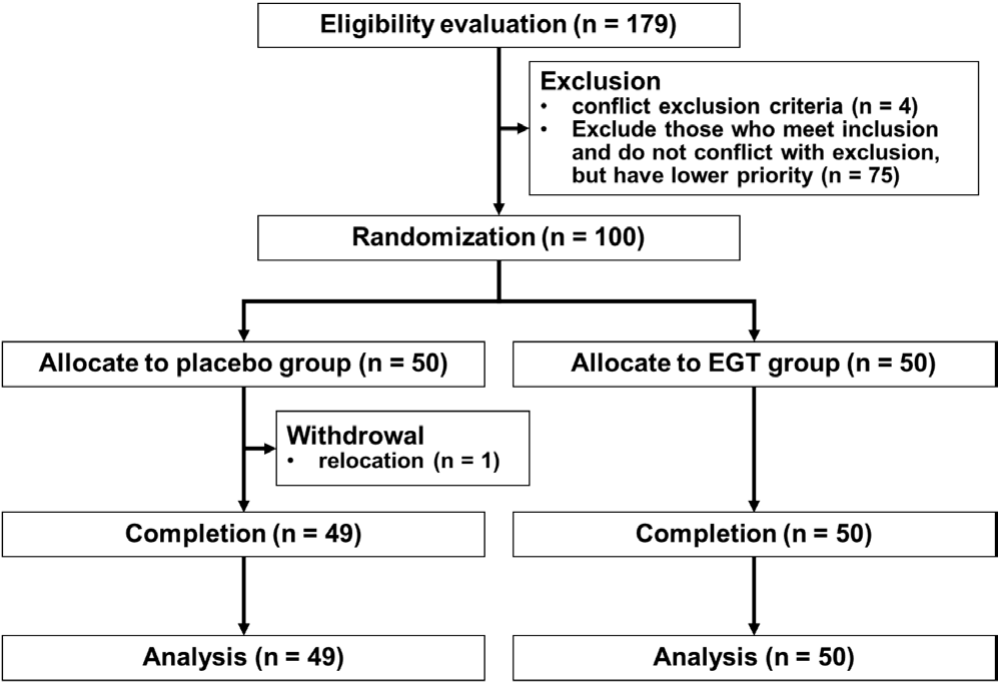
Flow diagram of participant recruitment for Study 2. One participant from the placebo group was withdrawn. In addition, participants who were ineligible for each measurement item were excluded according to the established criteria, and the results were described in each chapter.

In total, 100 participants (50 each in the EGT and placebo groups) participated in the study. One participant from the placebo group withdrew owing to relocation, resulting in a final count of 49 participants in the placebo group (Figure 3). Data from 92 participants from the OSA‐MA (45 in the EGT group and 47 in the placebo group) were obtained, excluding seven people who did not adequately complete the questionnaire. Data from one participant who did not undergo an MRI examination owing to fear were excluded from the BHQ results (50 in the intervention group and 48 in the control group). Of all participants, 51 were females (26 in the EGT group and 25 in the placebo group), and 49 were males (24 in the EGT group and 25 in the placebo group). The mean baseline characteristics were not significantly different between the groups (Table 3). Adverse events were evaluated in terms of the number of cases, number of occurrences, and incidence rates, and no significant differences were observed between the groups. There were no safety issues in conducting the study. Furthermore, no capsule intake of < 90% was observed.

**TABLE 3:** Baseline characteristics of the participants in Study 2.

	Placebo (*n* = 49)	Active (*n* = 50)	*p*
Male/female	24/25	24/26	> 0.99
Age (years)	53.5 ± 6.3	53.4 ± 6.0	0.94
Height (cm)	164.1 ± 8.1	164.2 ± 8.0	0.91
Weight (kg)	61.3 ± 11.1	61.6 ± 12.5	0.92
BMI (kg/m^2^)	22.7 ± 3.1	22.7 ± 3.5	> 0.99
PSQI‐total score	5.6 ± 2.2	5.7 ± 2.3	0.71
STAI‐state anxiety^a^	41.2 ± 8.5	41.7 ± 7.5	0.71
STAI‐trait anxiety^b^	44.1 ± 9.7	43.5 ± 9.7	0.76
Cognitrax‐NCI	98.3 ± 8.2	98.0 ± 8.8	0.87

Abbreviations: BMI, body mass index; NCI, neurocognitive index using Cognitrax; PSQI, Pittsburgh Sleep Quality Index; STAI, State–Trait Anxiety Inventory.

^a^ Placebo (*n* = 48), active (*n* = 50).

^b^ Placebo (*n* = 48), active (*n* = 48). Data are presented as means ± standard deviations.

#### 3.2.2 Plasma Levels of EGT

The plasma EGT levels increased 2.3‐fold in the active group following intervention compared with the baseline (from 4.07 to 9.38 μM, Figure 2d), whereas there was no significant change in the corresponding levels in the placebo group until Week 16 (from 3.67 to 3.27 μM). No difference was observed in the baseline plasma levels between the two groups. The observed plasma concentration of 8 mg/day after 16 weeks in Study 2 was 6.20% lower than that of the simulated value (Figure 2d).

#### 3.2.3 OSA‐MA Version

A between‐group comparison showed that the change in Factor IV (Refreshing) and Factor V (Sleep length) was significantly improved in the EGT group compared to that in the placebo group (Table 4). The “maintenance of sleep,” which is a component of Factor II (Initiation and maintenance of sleep), showed an improving between‐group trend. Within‐group changes in Factors IV (Refreshing) and V (Sleep length) significantly improved only in the EGT group. Factor II (Initiation and maintenance of sleep) showed an improving trend in the EGT group (*p* < 0.1), whereas Factor III (Frequent dreaming) was significantly worse in both groups (Table 4).

**TABLE 4:** Change in each factor of OSA‐MA.

OSA‐MA	Group	*N*	Baseline	Week 16	Δ values	*p*
Factor I (Sleepiness on rising)	Placebo	47	19.1 ± 5.1	18.9 ± 4.5	−0.2 ± 5.7	0.276
	EGT	45	17.3 ± 5.2	18.3 ± 6.4	1.0 ± 4.5	
Factor II (Initiation and maintenance of sleep)	Placebo	47	19.2 ± 4.9	18.7 ± 5.5	−0.5 ± 4.9	0.107
	EGT	45	18.0 ± 4.9	19.2 ± 5.6†	1.1 ± 4.5	
Factor III (Frequent dreaming)	Placebo	47	25.0 ± 5.2	23.3 ± 6.2*	−1.8 ± 5.4	0.922
	EGT	45	24.4 ± 4.9	22.8 ± 6.1*	−1.6 ± 5.3	
Factor IV (Refreshing)	Placebo	47	20.3 ± 5.1	20.2 ± 4.4	−0.1 ± 5.7	0.026
	EGT	45	18.5 ± 5.9	21.0 ± 6.1*	2.5 ± 5.2	
Factor V (Sleep length)	Placebo	47	19.6 ± 5.7	19.2 ± 4.9	−0.4 ± 5.5	0.007
	EGT	45	17.6 ± 5.0	20.3 ± 5.4*	2.7 ± 5.1	

*Note:* Data are presented as means ± standard errors. The paired *t*‐test was performed for intergroup comparisons from baseline. *p < 0.05, †*p* < 0.1; *p*‐values indicate differences between the groups for changes from baseline to Week 16. Two independent sample *t*‐tests were performed. EGT, ergothioneine; OSA‐MA, Oguri‐Shirakawa‐Azumi Sleep Inventory MA Version.

#### 3.2.4 Exploratory Items

There was no significant difference between the groups in the amount of change in the GM‐BHQ and FA‐BHQ scores over 16 weeks (Table S4). Meanwhile, the Curiosity of Embracing, CEI‐II, Specific, and Privacy significantly increased within the EGT group from baseline to Week 16, whereas it did not significantly change in the placebo group; however, there were no significant differences between the groups. In the between‐group comparison, only specific curiosity showed an improving trend (*p* < 0.1; Table S4).

#### 3.2.5 Blush‐Up of the PBPK Model and Sensitivity Analysis

**FIGURE 4:**
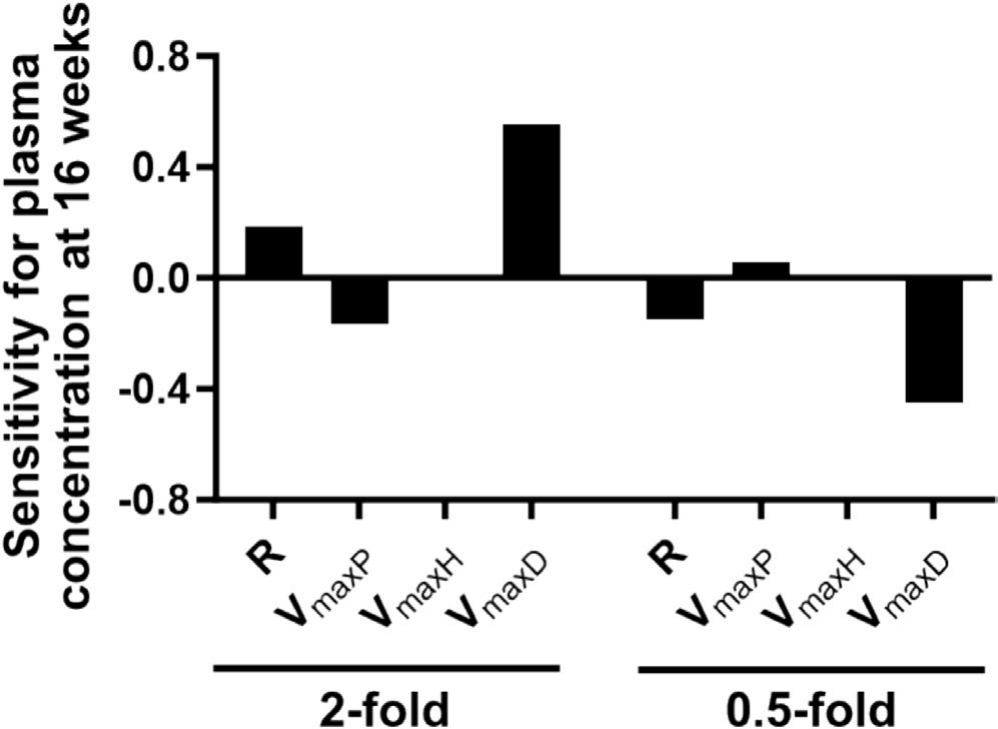
Sensitivity analysis for plasma EGT concentration after repeated oral ingestion. The simulation of the change in plasma EGT concentration at 16 weeks following repeated oral ingestion of EGT (8 mg/day) due to 2‐ and 0.5‐fold change in each parameter was conducted based on the PBPK model. Sensitivity, as shown on the ordinate, was calculated using Equation (2). The initial value for *C*_Plasma_ was set at 4.07 μM (the blank value at 8 mg/day), whereas that for *C*_RBC_ was set at 594 μM (the average of blank values at 5 mg/day and 10 mg/day). EGT, ergothioneine; PBPK, physiologically based pharmacokinetic; R, daily ingestion rate of EGT via diet; RBC, red blood cell; *V*_max_, maximum uptake rate.

Further fitting was performed to both plasma and RBC concentration profiles of EGT at 5, 8, 10, and 20 mg/day. The fitted lines closely overlapped with the data (Figure S2A–D). The optimized values of the three parameters (*R*, *V*_maxD_, and *V*_maxP_; Table S5) were slightly (1.12, 0.991, and 0.868 times, respectively) different from those obtained above (Table 1), while the CV values of the optimized parameters were within 8%–20% (Table S5). To clarify the most critical parameters affecting plasma EGT concentration, sensitivity analysis was conducted for the three optimized parameters and maximum uptake rate in the liver (*V*_maxH_). When these parameters were altered twofold, both *R* and *V*_maxD_ values provided a positive effect on plasma EGT concentration, whereas *V*_maxP_ provided a negative effect. No discernible effect of *V*_maxH_ was observed (Figure 4). In contrast, when the parameters were changed 0.5‐fold, both R and *V*_maxD_ values provided a negative effect on plasma EGT concentration, while *V*_maxP_ demonstrated a positive effect. No significant effect of *V*_maxH_ was observed (Figure 4).

### 3.3 Discussion

#### 3.3.1 Usefulness of PBPK Models in EGT and Other Food Compounds

Recently, several food‐derived ingredients have been developed for use as functional health foods. Food components generally exhibit milder effects over a longer consumption period compared to medicines. Therefore, one of the key points in developing health foods is to quantitatively evaluate the amount of active ingredients that should be ingested and the duration necessary to achieve the desired functionality. To answer these questions, clinical trials should be conducted to examine the biological response to the ingestion of food components over a long period. In drug development, the effective dosage and administration period are quantitatively predicted before conducting time‐consuming clinical trials, and the construction of a PBPK model is a useful approach for the prediction (Huang et al. 2013; Abouir et al. 2021). However, the understanding of the biodistribution of food components and the available approaches using such mathematical modeling to develop health foods is limited. The present study initially estimated the effective EGT plasma concentration in a limited number of individuals who ingested a relatively high amount of EGT over a short period, followed by the construction of a PBPK model for EGT to determine the appropriate dosage. This allowed us to determine the time required to reach an effective concentration at different doses (Table 2). Functionality was demonstrated at the optimized daily dose (8 mg for 16 weeks, Table 2), suggesting the validity of this strategy for dose estimation. The use of mathematical modeling in health food development represents a substantial advancement. However, this method may have several limitations, particularly in cases where the active ingredients are unknown or are extremely numerous. EGT possesses antioxidant effects, and its putative metabolites have been reported in humans; nonetheless, its plasma concentrations are low (Cheah et al. 2017). Thus, EGT itself could be an active ingredient enabling the estimation of effective intake based on the PBPK model. Furthermore, the site of EGT action could be the central nervous system (Ishimoto and Kato 2022), while the effective EGT dose was determined using the plasma EGT concentration in the present study. Although EGT exhibits brain distribution in mice, the extent of its distribution within the human brain remains largely unknown. While the PBPK model offers theoretical effectiveness in evaluating tissue concentrations, further studies are required to improve its reliability.

#### 3.3.2 Efficacy of EGT in Sleep Quality Improvement

In our study, we investigated the impact of EGT intake on sleep quality using the OSA‐MA scale, revealing significant improvements in Factor IV (Refreshing), Factor V (Sleep length), and a Factor II component (Sleep maintenance), showing a tendency for improvement (*p* < 0.1) in healthy individuals (Table 4). Conversely, Factors I (Sleepiness on rising), II (Initiation and maintenance), and III (Frequent dreaming) did not exhibit significant improvements (Table 4). Factors such as sleepiness upon waking and frequent dreaming may be influenced by external factors, such as the ambient environment (e.g., brightness) and experiences from the previous day. Therefore, to accurately evaluate their effects, it may be necessary to assess them in a more controlled testing environment.

In the previous study by Katsube et al. (2022), enhancements in “sleep difficulty” were noted on the PSQI‐J, indicating an improvement in subjective sleep quality. This observation aligns well with the substantial improvement trend observed in “Sleep maintenance (Factor II component)” on the OSA‐MA scale in our current investigation. In addition, our study demonstrated notable enhancements in Factor IV (Refreshing) and Factor V (Sleep length) on the OSA‐MA, aspects that were not evaluated by the PSQI‐J. The comprehensive nature of the OSA‐MA, designed with multiple items to assess mood at the time of completion, resonates with previous reports highlighting improvements in waking mood following the consumption of food ingredients (Miyake et al. 2014; Morikawa et al. 2019; Nakashima et al. 2020; Ozeki et al. 2004, 2008; Uesaki et al. 2016, 2018; Umigai et al. 2018).

In the previous study, EGT was found to increase the N2 stage and decrease the N1 stage in NREM sleep, along with reducing the frequency of WASO, indicating improvements in objective sleep quality (Katsube et al. 2022). However, there are no existing reports directly correlating these factors with the OSA‐MA factor. In contrast, there is a study suggesting that a different objective sleep indicator, sleep duration, correlates with Factors II (Initiation and maintenance of sleep), IV (Refreshing), and V (Sleep length; Okamura et al. 2020). Although the improvements observed in Factor IV (Refreshing), Factor V (Sleep length), and the component of Factor II (Sleep maintenance) with EGT intake seem reasonable (Table 4), further discussion is difficult because sleep duration was not improved by EGT intake in the previous study.

Overall, it is possible that EGT improved sleep quality through a similar mechanism as outlined in the previous study by Katsube et al. (2022). This mechanism may involve the suppression of α‐3‐hydroxy‐5‐methyl‐4‐isoxazole propionic acid receptor due to decreased glutamate levels, reduction of stress hormones, and attenuation of oxidative stress and inflammation. Notably, the concentrations of EGT achieved in our study (9.37 μM) closely approximated the target plasma concentrations (9.51 μM), further supporting the likelihood of similar improvements in subjective sleep quality as described earlier.

#### 3.3.3 Effects of EGT on Curiosity

Significant differences in curiosity were observed before and after intake within the EGT group (Table S4). However, no significant differences were noted in between‐group comparisons (Table S4). Therefore, additional studies are required to clarify the effects of EGT on curiosity. The potential augmentation of curiosity following EGT intake would represent a novel discovery. Notably, previous research has demonstrated improved novel object recognition in mice following EGT administration (Nakamichi et al. 2021). Nevertheless, this study holds promise for further advancement owing to the following reasons: (1) this study only evaluated curiosity, whereas the previous study reflected curiosity and learning; (2) this study evaluated human curiosity, whereas no studies have tested this aspect in mice; and (3) our evaluation encompassed various facets of curiosity, including epistemic and interpersonal curiosity, as well as curiosity and exploration inventory, which encompasses curiosity concerning knowledge and human interactions. This broader scope contrasts with previous studies that primarily evaluated primitive curiosity related to a novel object.

#### 3.3.4 Effects of EGT on Other Items

There were no significant differences in FA‐BHQ and GM‐BHQ scores between the groups (Section 3.2.4). However, in this study, the FA‐BHQ scores at baseline were significantly different between the groups, which may have prevented a valid assessment. In contrast, GM‐BHQ did not significantly differ between the groups at baseline. Unexpectedly, GM‐BHQ did not decline in the placebo group but improved by 0.12 points. Given the 0.6‐point decrease over 1 year in the previous report (Nemoto et al. 2017), a decrease of approximately 0.2 points over 16 weeks can be expected. We expected EGT to possess inhibitory effects on age‐related decreases in the GM‐BHQ scores; however, the present study was performed under an unsuitable condition for this objective. Therefore, it is recommended that the effects of FA‐BHQ and GM‐BHQ be re‐evaluated with a revised study design, incorporating changes in the selection criteria for participants and the duration of the study period.

#### 3.3.5 Fitting Validity

In this study, the PBPK model construction was limited by the availability of limited experimental data, primarily comprising measured blood and plasma concentrations. Therefore, only three parameters were optimized, and many parameters were fixed based on information obtained from mouse studies. Therefore, the validity of the obtained model must be carefully verified. For example, the R‐value was estimated to be 7.48 mg (1.36 μmol/70 kg body weight/h; Table 1). The estimated daily intake of dietary EGT in France, Italy, and the United States is approximately 2.24–3.57 mg, 4.69–17.1 mg, and 1.12–10.7 mg, respectively (Ramirez‐Martinez et al. 2016). These estimations suggest that the modeled dietary EGT levels are within realistic ranges. Meanwhile, it was assumed that absorption from the small intestine is complete in the absorption process (Figure 2). However, the rate of EGT absorption in the small intestine via OCTN1 may be altered by diseases or aging. Actually, decreased blood EGT concentrations in chronic kidney disease can be attributed to decreased absorption from the small intestine (Shinozaki et al. 2017). Therefore, the PBPK model constructed in the present study should be used with caution.

#### 3.3.6 Sensitivity Analysis

The sensitivity analysis conducted on varying pharmacokinetic parameters identified renal reabsorption (*V*_maxD_) and dietary EGT intake (*R*) as the most influential factors affecting plasma EGT concentrations (Figure 4). Notably, studies by Cheah et al. (2016) and Shinozaki et al. (2017) have reported lower plasma EGT concentrations in older individuals and elderly patients with cognitive decline, respectively. Thus, large interindividual variability may exist in plasma EGT concentrations, and this may be governed at least by daily EGT intake and renal reabsorption.

In contrast, given the potential health benefits associated with EGT supplementation, individuals with lower plasma EGT levels may particularly benefit from EGT‐enriched dietary interventions. Therefore, it is important to evaluate the dietary blood EGT concentrations in the target population. In the future, a more quantitative clarification of the relationship between renal function, EGT intake, and the disposition of EGT in the body may facilitate the prediction of plasma EGT concentrations in specific population subsets.

Conversely, the sensitivity analysis revealed a lower sensitivity of the parameter representing hepatic uptake (*V*_maxH_; Figure 4). As hepatic uptake is closely aligned with the blood‐flow rate, changes in *V*_maxH_ do not significantly affect the ability of the entire organ to take up EGT, and hepatic uptake does not directly influence the disappearance of EGT from the body.

#### 3.3.7 Peak in Plasma EGT Concentration After a Single Oral Administration

In mice, the plasma concentrations of [^3^H]EGT exhibit a gradual increase over time, reaching a peak at approximately 4 h post administration, as reported by Sugiura et al. (2010). In contrast, *Octn1* gene‐deficient mice demonstrate an immediate peak plasma concentration shortly after oral administration, followed by rapid clearance from the bloodstream (Kato et al. 2010). This discrepancy is attributed to the efficient uptake of EGT absorbed in the gastrointestinal tract by the liver via OCTN1, with clearance rates comparable to the hepatic plasma flow rate (Sugiura et al. 2010).

In our study, the simulation of plasma EGT concentrations based on the constructed PBPK model (Figure S1) did not exhibit clear peaks. This absence of peaks is attributed to the fact that the clearance of EGT uptake in the liver was set to a value closely aligned with the hepatic plasma flow rate. However, the immediate trend in plasma EGT concentration following administration in humans remains unknown and needs to be clarified by further studies.

The direct evaluation of organ EGT uptake in humans presents considerable challenges. Consequently, in our study, organ *V*_max_ values were estimated by fitting, assuming a proportionality to OCTN1 RNA expression levels. Nevertheless, it is essential to acknowledge that such an assumption may not accurately represent the true relationship between the *V*_max_ values and *Octn1* gene expression. This is because the *V*_max_ values may not solely depend on *Octn1* gene expression, considering the potential influence of accessory proteins on the function and/or expression of this transporter (Gisler et al. 2003; Sugiura et al. 2006). Therefore, further validation of tissue uptake of EGT in humans is necessary to refine our understanding of its pharmacokinetics.

#### 3.3.8 Limitations of This Study

Study 2 was aimed at testing the effect of EGT 8 mg/day on sleep quality but was submitted as an exploratory study with BHQ as the primary endpoint, assuming that there was an improvement in sleep quality. Considering that the conditions of EGT intake and the tests that evaluated sleep quality are different from those reported by Katsube et al. (2022), it would be desirable to verify its effectiveness in improving sleep quality in a clinical trial. In addition, the PBPK model constructed in the present study did not consider the observed data of plasma EGT level during daily food ingestion or those after placebo administration to simplify the modeling process. EGT is ingested by daily life, which may show quite large interindividual variability, although the data chasing EGT profiles during daily life are not currently available. The PBPK model in the present study tentatively included R value as the daily ingestion rate of EGT, but the reliability of this parameter might be limited. Thus, the current PBPK model may still be a preliminary one, and incorporating the change in EGT due to its daily ingestion from food is challenging and should be performed in the future.

## 4 Conclusion

We proposed a new approach to estimate the minimum efficacious doses of a food ingredient using a PBPK model. The study was conducted in two parts: Study 1 involved the optimization of the PBPK model based on plasma and blood EGT concentration profiles. Study 2 evaluated the effect of the optimized dosing regimens on sleep quality, guided by the PBPK model analysis. Additionally, the improvement in sleep quality was corroborated using the OSA‐MA, in accordance with the hypothesized effect. This methodology may be applicable to various food ingredients in the future.

**Nomenclature**
BHQ: Brain Healthcare Quotient
BMI: body mass index
*C*
: EGT concentration
CEI‐II: Curiosity and Exploration Inventory‐II
CV: coefficient of variation
EGT: ergothioneine
FA: fractional anisotropy
GM: gray matter
IS: internal standard
*K*_m_
: Michaelis constant
MRI: magnetic resonance imaging
NCI: neurocognitive index
NREM: non‐rapid eye movement
OSA‐MA: Oguri‐Shirakawa‐Azumi Sleep Inventory MA Version
PBPK: physiologically based pharmacokinetic
PS_eff_
: intrinsic efflux clearance
PSQI: Pittsburgh Sleep Quality Index
*R*
: daily ingestion rate of EGT via diet
RBC: red blood cell
STAI: State–Trait Anxiety Inventory
*V*_max_
: maximum uptake rate
WASO: waking after sleep onset

## Author Contributions

**Hitoshi Okumura:** conceptualization (equal), data curation (equal), formal analysis (equal), investigation (equal), methodology (equal), project administration (equal), writing – original draft (equal). **Yudai Araragi:** data curation (equal), formal analysis (equal), investigation (equal), methodology (equal), software (equal), writing – original draft (equal). **Kentaro Nishioka:** conceptualization (equal), methodology (equal), writing – review and editing (equal). **Reiya Yamashita:** formal analysis (equal), methodology (equal). **Toshihide Suzuki:** project administration (equal), writing – review and editing (equal). **Hiroshi Watanabe:** conceptualization (equal), funding acquisition (equal), project administration (equal), supervision (equal), writing – review and editing (equal). **Yukio Kato:** formal analysis (equal), investigation (equal), methodology (equal), software (equal), supervision (equal), writing – review and editing (equal). **Norihito Murayama:** conceptualization (equal), funding acquisition (equal), project administration (equal), resources (equal), supervision (equal), writing – review and editing (equal).

## Conflicts of Interest

H.O., K.N., H.W., T.S., and N.M. were employed by Suntory Global Innovation Center Ltd. The remaining authors declare that the research was conducted in the absence of any commercial or financial relationships that could be construed as a potential conflicts of interest.

## Supporting information


Data S1.

## Data Availability

The dataset supporting the conclusions of this article is included within the article and its Supporting Information.
