# Supplementary material for: Estimation and Validation of an Effective Ergothioneine Dose for Improved Sleep Quality Using Physiologically Based Pharmacokinetic Model
Source: Food Sci Nutr. 2025 Jun 5;13(6):e70382. doi: 10.1002/fsn3.70382 (PMC12138820; doi:10.1002/fsn3.70382)

Supporting Information

# Supplementary data

## Nomenclature

$C_{d}$, EGT concentration in duct; $C_{P}$, EGT concentration in plasma; $C_{\mathrm{RBC}}$, EGT concentration in RBC; $C_{T}$, EGT concentration in tissue intracellular space; $C_{TE}$, EGT concentration in tissue extracellular space; EGT, ergothioneine; $GER$, gastric emptying rate; $GFR$, glomerular filtration rate; k_1_, rate constant for RBC differentiation; k_b_, rate constant for RBC degradation; $K_{m}$, Michaelis constant (OCTN1); $K_{pT}$, tissue-to-plasma concentration ratio; ${PS}_{effT}$, intrinsic efflux clearance in tissue; RBC, red blood cell; $Q_{t}$, tissue plasma flow rate; $Q_{u}$, urinary flow rate; $R$, daily ingestion rate of EGT via diet; $R_{T}$, ratio of Vmax values in tissue; $V_{maxD}$, maximum uptake rate in renal reabsorption; $V_{maxP}$, maximum uptake rate in \ precursor; $V_{maxT}$, maximum uptake rate in tissue; $V_{P}$, volumes of plasma; $V_{RBC}$, volumes of RBC; $V_{t}$,volumes of tissue intracellular space; $V_{te}$, volumes of tissue extracellular space; ${}X_{i}$, EGT amount in gastrointestinal tract; $X_{pre}$, EGT amount in RBC precursor.

## Differential equations

Gastrointestinal tract compartment

$$\frac{dX_{i}}{\mathrm{dt}}=R-GER\times X_{i}$$

Plasma compartment

$$V_{p}\times\frac{dC_{p}}{\mathrm{dt}}=-\left( Q_{a}+Q_{h}+Q_{m}+Q_{s} \right)\times C_{p}+Q_{a}\times C_{\mathrm{AE}}+Q_{h}\times C_{HE, 5}+Q_{m}\times C_{\mathrm{ME}}+Q_{s}\times C_{\mathrm{SE}}-\frac{V_{\mathrm{maxP}}\times C_{P}}{K_{m}+C_{P}}+k_{b}\times C_{\mathrm{RBC}}\times V_{\mathrm{RBC}}-GFR\times C_{P}+\frac{V_{\mathrm{maxD}}\times C_{d}}{K_{m}+C_{d}}$$

Tissue extracellular space compartments (adipose, muscle, and skin)

$$V_{\mathrm{te}}\times\frac{dC_{\mathrm{TE}}}{\mathrm{dt}}=Q_{t}\times(C_{P}-C_{\mathrm{TE}})-\frac{V_{\mathrm{maxT}}\times C_{\mathrm{TE}}}{K_{m}+C_{\mathrm{TE}}}+\mathrm{PS}_{\mathrm{effT}}\times C_{T}$$

Tissue intracellular space compartments (adipose, muscle, and skin)

$$V_{t}\times\frac{dC_{T}}{\mathrm{dt}}=\frac{V_{\mathrm{maxT}}\times C_{\mathrm{TE}}}{K_{m}+C_{\mathrm{TE}}}-\mathrm{PS}_{\mathrm{effT}}\times C_{T}$$

Liver extracellular space (HE, i = 1–5) compartments

$$\frac{V_{\mathrm{he}}}{5}\times\frac{dC_{HE, i}}{\mathrm{dt}}=GER\times X_{i}+Q_{h}\times\left( C_{P}-C_{HE, i} \right)-\frac{V_{\mathrm{maxH}}\times C_{HE, i}}{K_{m}+C_{HE, i}}+\mathrm{PS}_{\mathrm{effH}}\times C_{H, i} :(i=1)$$

$$\frac{V_{\mathrm{he}}}{5}\times\frac{dC_{HE, i}}{\mathrm{dt}}=Q_{h}\times\left( C_{HE, i-1}-C_{HE, i} \right)-\frac{V_{\mathrm{maxH}}\times C_{HE, i}}{K_{m}+C_{HE, i}}+\mathrm{PS}_{\mathrm{effH}}\times C_{H, i} :(i=2-5)$$

Liver intracellular space (H, i = 1–5) compartments

$$\frac{V_{\mathrm{hc}}}{5}\times\frac{dC_{H, i}}{\mathrm{dt}}=\frac{V_{\mathrm{maxH}}\times C_{HE, i}}{K_{m}+C_{HE, i}}-\mathrm{PS}_{\mathrm{effH}}\times C_{H, i}$$

Renal duct compartment

$$V_{d}\times\frac{dC_{d}}{\mathrm{dt}}=GFR\times C_{P}-\frac{V_{\mathrm{maxD}}\times C_{d}}{K_{m}+C_{d}}-Q_{u}\times C_{d}$$

RBC precursor compartment

$$\frac{dX_{\mathrm{pre}}}{\mathrm{dt}}=\frac{V_{\mathrm{maxP}}\times C_{P}}{K_{m}+C_{P}}-k_{1}\times X_{\mathrm{pre}}$$

RBC compartment

$$V_{\mathrm{RBC}}\times\frac{dC_{\mathrm{RBC}}}{\mathrm{dt}}=k_{1}\times X_{\mathrm{pre}}-k_{b}\times C_{\mathrm{RBC}}\times V_{\mathrm{RBC}}$$

Other equations

$$V_{\mathrm{maxS}}=R_{S}\times V_{\mathrm{maxH}}$$

$$V_{\mathrm{maxM}}=R_{M}\times V_{\mathrm{maxH}}$$

$$V_{\mathrm{maxA}}=R_{A}\times V_{\mathrm{maxH}}$$

$$\mathrm{PS}_{\mathrm{effH}}=\frac{V_{\mathrm{maxH}}}{K_{m}}\times\frac{1}{K_{\mathrm{pH}}}$$

$$\mathrm{PS}_{\mathrm{effS}}=R_{S}\times\frac{V_{\mathrm{maxH}}}{K_{m}}\times\frac{1}{K_{\mathrm{pS}}}$$

$$\mathrm{PS}_{\mathrm{effM}}=R_{M}\times\frac{V_{\mathrm{maxH}}}{K_{m}}\times\frac{1}{K_{\mathrm{pM}}}$$

$$\mathrm{PS}_{\mathrm{effA}}=R_{A}\times\frac{V_{\mathrm{maxH}}}{K_{m}}\times\frac{1}{K_{\mathrm{pA}}}$$

## Estimation of initial values

The above mass-balance equations include 19 unknown initial values of EGT concentrations and amounts in tissue compartments ($X_{i}$, $C_{HE, 1}$, $C_{HE, 2}$, $C_{HE, 3}$, $C_{HE, 4}$, $C_{HE, 5}$, $C_{H, 1}$, $C_{H, 2}$, $C_{H, 3}$, $C_{H, 4}$, $C_{H, 5}$, $C_{\mathrm{AE}}$, $C_{A}$, $C_{\mathrm{ME}}$, $C_{M}$, $C_{\mathrm{SE}}$, $C_{S}$, $X_{\mathrm{pre}}$, and $C_{d}$). These initial values were numerically calculated as follows, assuming steady-state conditions:

$$X_{i (0)}= \frac{R}{\mathrm{GER}}$$

$$C_{HE, 1 (0)}=\frac{R}{\mathrm{Qh}}+C_{P (0)}$$

$$C_{HE, 2 (0)}=\frac{R}{\mathrm{Qh}}+C_{P (0)}$$

$$C_{HE, 3 (0)}=\frac{R}{\mathrm{Qh}}+C_{P (0)}$$

$$C_{HE, 4 (0)}=\frac{R}{\mathrm{Qh}}+C_{P (0)}$$

$$C_{HE, 5 (0)}=\frac{R}{\mathrm{Qh}}+C_{P (0)}$$

$$C_{H, 1 \left( 0 \right)}=\frac{V_{\mathrm{maxH}}\times\left( \frac{R}{\mathrm{Qh}}+C_{P \left( 0 \right)} \right)}{K_{m}+\frac{R}{\mathrm{Qh}}+C_{P \left( 0 \right)}}\times\frac{1}{\mathrm{PS}_{\mathrm{effH}}}$$

$$C_{H, 2 \left( 0 \right)}=\frac{V_{\mathrm{maxH}}\times\left( \frac{R}{\mathrm{Qh}}+C_{P \left( 0 \right)} \right)}{K_{m}+\frac{R}{\mathrm{Qh}}+C_{P \left( 0 \right)}}\times\frac{1}{\mathrm{PS}_{\mathrm{effH}}}$$

$$C_{H, 3 \left( 0 \right)}=\frac{V_{\mathrm{maxH}}\times\left( \frac{R}{\mathrm{Qh}}+C_{P \left( 0 \right)} \right)}{K_{m}+\frac{R}{\mathrm{Qh}}+C_{P \left( 0 \right)}}\times\frac{1}{\mathrm{PS}_{\mathrm{effH}}}$$

$$C_{H, 4 \left( 0 \right)}=\frac{V_{\mathrm{maxH}}\times\left( \frac{R}{\mathrm{Qh}}+C_{P \left( 0 \right)} \right)}{K_{m}+\frac{R}{\mathrm{Qh}}+C_{P \left( 0 \right)}}\times\frac{1}{\mathrm{PS}_{\mathrm{effH}}}$$

$$C_{H, 5 \left( 0 \right)}=\frac{V_{\mathrm{maxH}}\times\left( \frac{R}{\mathrm{Qh}}+C_{P \left( 0 \right)} \right)}{K_{m}+\frac{R}{\mathrm{Qh}}+C_{P \left( 0 \right)}}\times\frac{1}{\mathrm{PS}_{\mathrm{effH}}}$$

$$C_{AE (0)}=C_{P (0)}$$

$$C_{A \left( 0 \right)}=\frac{V_{\mathrm{maxA}}\times C_{P \left( 0 \right)}}{K_{m}+C_{P \left( 0 \right)}}\times\frac{1}{\mathrm{PS}_{\mathrm{effA}}}$$

$$C_{ME (0)}=C_{P (0)}$$

$$C_{M \left( 0 \right)}=\frac{V_{\mathrm{maxM}}\times C_{P \left( 0 \right)}}{K_{m}+C_{P \left( 0 \right)}}\times\frac{1}{\mathrm{PS}_{\mathrm{effM}}}$$

$$C_{SE (0)}=C_{P (0)}$$

$$C_{S \left( 0 \right)}=\frac{V_{\mathrm{maxS}}\times C_{P \left( 0 \right)}}{K_{m}+C_{P \left( 0 \right)}}\times\frac{1}{\mathrm{PS}_{\mathrm{effS}}}$$

$$X_{pre (0)}= \frac{V_{\mathrm{maxP}}\times C_{P \left( 0 \right)}}{K_{m}+C_{P \left( 0 \right)}}\times\frac{1}{k_{1}}$$

$$C_{d \left( 0 \right)}=\frac{R}{Q_{u}}$$

## Actual model code used in Napp software

@At 0:

y1=Cp0,

y2=R/ka+Dose,

y3=R/Qh+Cp0,

y4=VmaxH*(R/Qh+Cp0)/(Km+R/Qh+Cp0)/PSeffH,

y5=R/Qh+Cp0,

y6=VmaxH*(R/Qh+Cp0)/(Km+R/Qh+Cp0)/PSeffH,

y7=R/Qh+Cp0,

y8=VmaxH*(R/Qh+Cp0)/(Km+R/Qh+Cp0)/PSeffH,

y9=R/Qh+Cp0,

y10=VmaxH*(R/Qh+Cp0)/(Km+R/Qh+Cp0)/PSeffH,

y11=R/Qh+Cp0,

y12=VmaxH*(R/Qh+Cp0)/(Km+R/Qh+Cp0)/PSeffH,

y13=Cp0,

y14=VmaxS*Cp0/(Km+Cp0)/PSeffS,

y15=Cp0,

y16=VmaxM*Cp0/(Km+Cp0)/PSeffM,

y17=Cp0,

y18=VmaxA*Cp0/(Km+Cp0)/PSeffA,

y19=VmaxP*Cp0/(Km+Cp0)/k1,

y20=Crbc0,

y21=R/Qu,

@At 24:

y2 +=Dose,

@At 48:

y2 +=Dose,

@At 72:

y2 +=Dose,

@At 96:

y2 +=Dose,

@At 120:

y2 +=Dose,

@At 144:

y2 +=Dose,

@At 168:

y2 +=Dose,

@At 192:

y2 +=Dose,

@At 216:

y2 +=Dose,

@At 240:

y2 +=Dose,

@At 264:

y2 +=Dose,

@At 288:

y2 +=Dose,

@At 312:

y2 +=Dose,

@At 336:

y2 +=Dose,

@At 360:

y2 +=Dose,

@At 384:

y2 +=Dose,

@At 408:

y2 +=Dose,

@At 432:

y2 +=Dose,

@At 456:

y2 +=Dose,

@At 480:

y2 +=Dose,

@At 504:

y2 +=Dose,

@At 528:

y2 +=Dose,

@At 552:

y2 +=Dose,

@At 576:

y2 +=Dose,

@At 600:

y2 +=Dose,

@At 624:

y2 +=Dose,

@At 648:

y2 +=Dose,

@At 672:

y2 +=Dose,

@At 696:

y2 +=Dose,

@At 720:

y2 +=Dose,

@At 744:

y2 +=Dose,

@At 768:

y2 +=Dose,

@At 792:

y2 +=Dose,

@At 816:

y2 +=Dose,

@At 840:

y2 +=Dose,

@At 864:

y2 +=Dose,

@At 888:

y2 +=Dose,

@At 912:

y2 +=Dose,

@At 936:

y2 +=Dose,

@At 960:

y2 +=Dose,

@At 984:

y2 +=Dose,

@At 1008:

y2 +=Dose,

@At 1032:

y2 +=Dose,

@At 1056:

y2 +=Dose,

@At 1080:

y2 +=Dose,

@At 1104:

y2 +=Dose,

@At 1128:

y2 +=Dose,

@At 1152:

y2 +=Dose,

@At 1176:

y2 +=Dose,

@At 1200:

y2 +=Dose,

@At 1224:

y2 +=Dose,

@At 1248:

y2 +=Dose,

@At 1272:

y2 +=Dose,

@At 1296:

y2 +=Dose,

@At 1320:

y2 +=Dose,

@At 1344:

y2 +=Dose,

@At 1368:

y2 +=Dose,

@At 1392:

y2 +=Dose,

@At 1416:

y2 +=Dose,

@At 1440:

y2 +=Dose,

@At 1464:

y2 +=Dose,

@At 1488:

y2 +=Dose,

@At 1512:

y2 +=Dose,

@At 1536:

y2 +=Dose,

@At 1560:

y2 +=Dose,

@At 1584:

y2 +=Dose,

@At 1608:

y2 +=Dose,

@At 1632:

y2 +=Dose,

@At 1656:

y2 +=Dose,

@At 1680:

y2 +=Dose,

@At 1704:

y2 +=Dose,

@At 1728:

y2 +=Dose,

@At 1752:

y2 +=Dose,

@At 1776:

y2 +=Dose,

@At 1800:

y2 +=Dose,

@At 1824:

y2 +=Dose,

@At 1848:

y2 +=Dose,

@At 1872:

y2 +=Dose,

@At 1896:

y2 +=Dose,

@At 1920:

y2 +=Dose,

@At 1944:

y2 +=Dose,

@At 1968:

y2 +=Dose,

@At 1992:

y2 +=Dose,

@At 2016:

y2 +=Dose,

@At 2040:

y2 +=Dose,

@At 2064:

y2 +=Dose,

@At 2088:

y2 +=Dose,

@At 2112:

y2 +=Dose,

@At 2136:

y2 +=Dose,

@At 2160:

y2 +=Dose,

@At 2184:

y2 +=Dose,

@At 2208:

y2 +=Dose,

@At 2232:

y2 +=Dose,

@At 2256:

y2 +=Dose,

@At 2280:

y2 +=Dose,

@At 2304:

y2 +=Dose,

@At 2328:

y2 +=Dose,

@At 2352:

y2 +=Dose,

@At 2376:

y2 +=Dose,

@At 2400:

y2 +=Dose,

@At 2424:

y2 +=Dose,

@At 2448:

y2 +=Dose,

@At 2472:

y2 +=Dose,

@At 2496:

y2 +=Dose,

@At 2520:

y2 +=Dose,

@At 2544:

y2 +=Dose,

@At 2568:

y2 +=Dose,

@At 2592:

y2 +=Dose,

@At 2616:

y2 +=Dose,

@At 2640:

y2 +=Dose,

@At 2664:

y2 +=Dose,

@At 2688:

y2 +=Dose,

@From 0:

y1’=(-(Qh+Qs+Qm+Qa)*y1+Qh*y11+Qs*y13+Qm*y15+Qa*y17-VmaxP*y1/(Km+y1)+kb*y20*Vrbc-GFR*y1+Vmaxd*y21/(Km+y21))/Vp,

y2’=R-GER*y2,

y3’=5*(GER*y2+Qh*(y1-y3)-VmaxH*y3/(Km+y3)/5+PSeffH*y4/5)/Vhe,

y4’=5*(VmaxH*y3/(Km+y3)/5-PSeffH*y4/5)/Vh,

y5’=5*(Qh*(y3-y5)-VmaxH*y5/(Km+y5)/5+PSeffH*y6/5)/Vhe,

y6’=5*(VmaxH*y5/(Km+y5)/5-PSeffH*y6/5)/Vh,

y7’=5*(Qh*(y5-y7)-VmaxH*y7/(Km+y7)/5+PSeffH*y8/5)/Vhe,

y8’=5*(VmaxH*y7/(Km+y7)/5-PSeffH*y8/5)/Vh,

y9’=5*(Qh*(y7-y9)-VmaxH*y9/(Km+y9)/5+PSeffH*y10/5)/Vhe,

y10’=5*(VmaxH*y9/(Km+y9)/5-PSeffH*y10/5)/Vh,

y11’=5*(Qh*(y9-y11)-VmaxH*y11/(Km+y11)/5+PSeffH*y12/5)/Vhe,

y12’=5*(VmaxH*y11/(Km+y11)/5-PSeffH*y12/5)/Vh,

y13’=(Qs*(y1-y13)-VmaxS*y13/(Km+y13)+PSeffS*y14)/Vse,

y14’=(VmaxS*y13/(Km+y13)-PSeffS*y14)/Vs,

y15’=(Qm*(y1-y15)-VmaxM*y15/(Km+y15)+PSeffM*y16)/Vme,

y16’=(VmaxM*y15/(Km+y15)-PSeffM*y16)/Vm,

y17’=(Qa*(y1-y17)-VmaxA*y17/(Km+y17)+PSeffA*y18)/Vae,

y18’=(VmaxA*y17/(Km+y17)-PSeffA*y18)/Va,

y19’=VmaxP*y1/(Km+y1)-k1*y19,

y20’=(k1*y19-kb*y20*Vrbc)/Vrbc,

y21’=(GFR*y1-Vmaxd*y21/(Km+y21)-Qu*y21)/Vd,

*Preparative calculation*

PSeffH=VmaxH/Km/(Kph),

PSeffS=Rs*VmaxH/Km/(Kps),

PSeffM=Rm*VmaxH/Km/(Kpm),

PSeffA=Ra*VmaxH/Km/(Kpa),

VmaxS=Rs*VmaxH,

VmaxM=Rm*VmaxH,

VmaxA=Ra*VmaxH,

# Supplementary Figures and Tables

## Supplementary Figures


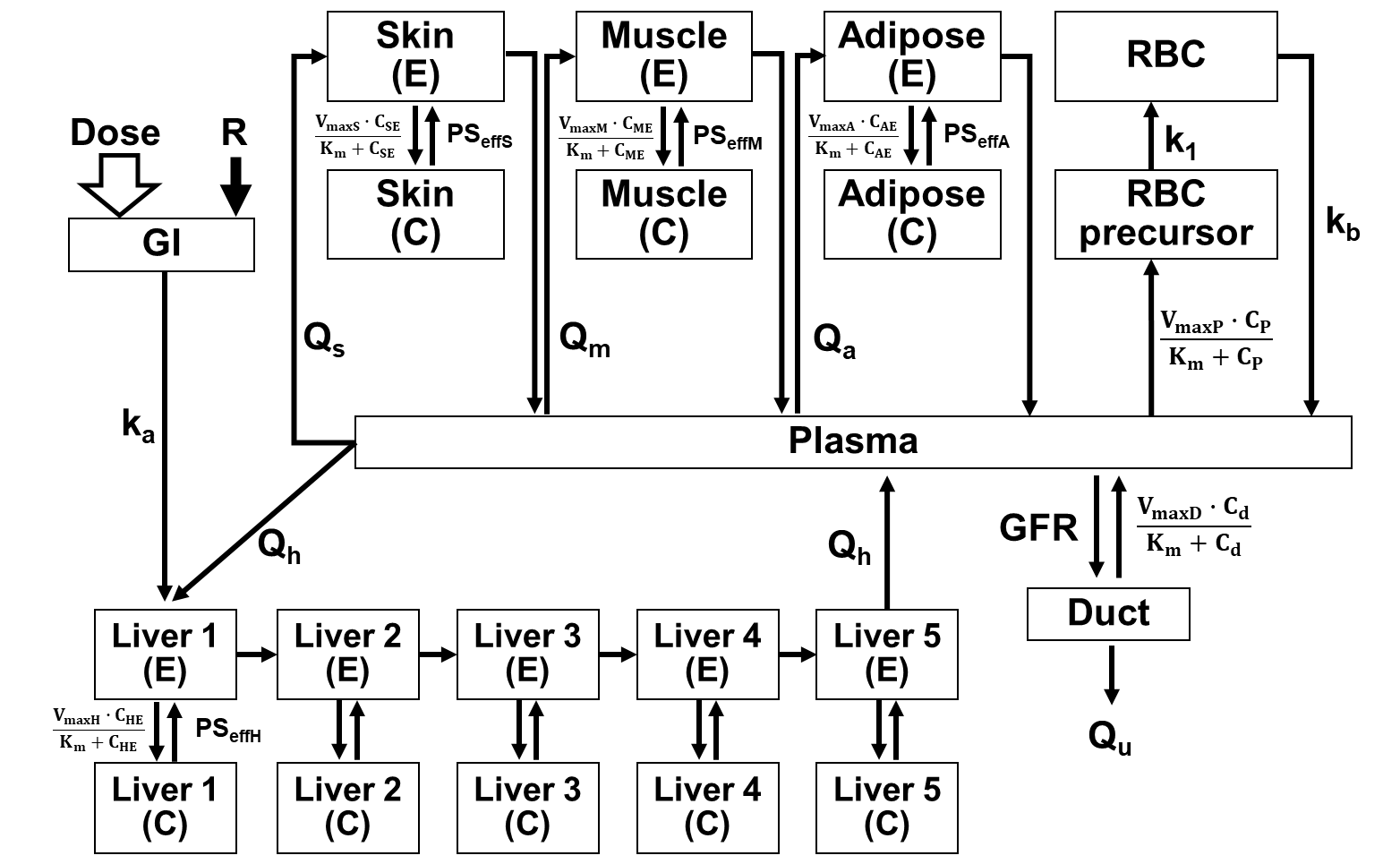


**SUPPLEMENTARY FIGURE 1** PBPK model for repeated oral administration of EGT in humans. (C), intracellular space in each tissue; (E), extracellular space in each tissue; C, EGT concentration; Duct, renal tubule duct; EGT, ergothioneine; GFR, glomerular filtration rate; GI, gastrointestinal tract; k_1_, rate constant for RBC differentiation; k_a_, absorption rate constant; k_b_, rate constant for RBC degradation; K_m_, Michaelis constant; PBPK, physiologically-based pharmacokinetic; PS_eff_, intrinsic efflux clearance; Q, plasma flow rate; Q_u_, urinary flow rate; R, daily ingestion rate of EGT via diet; RBC, red blood cells; V_max_, maximum uptake rate.


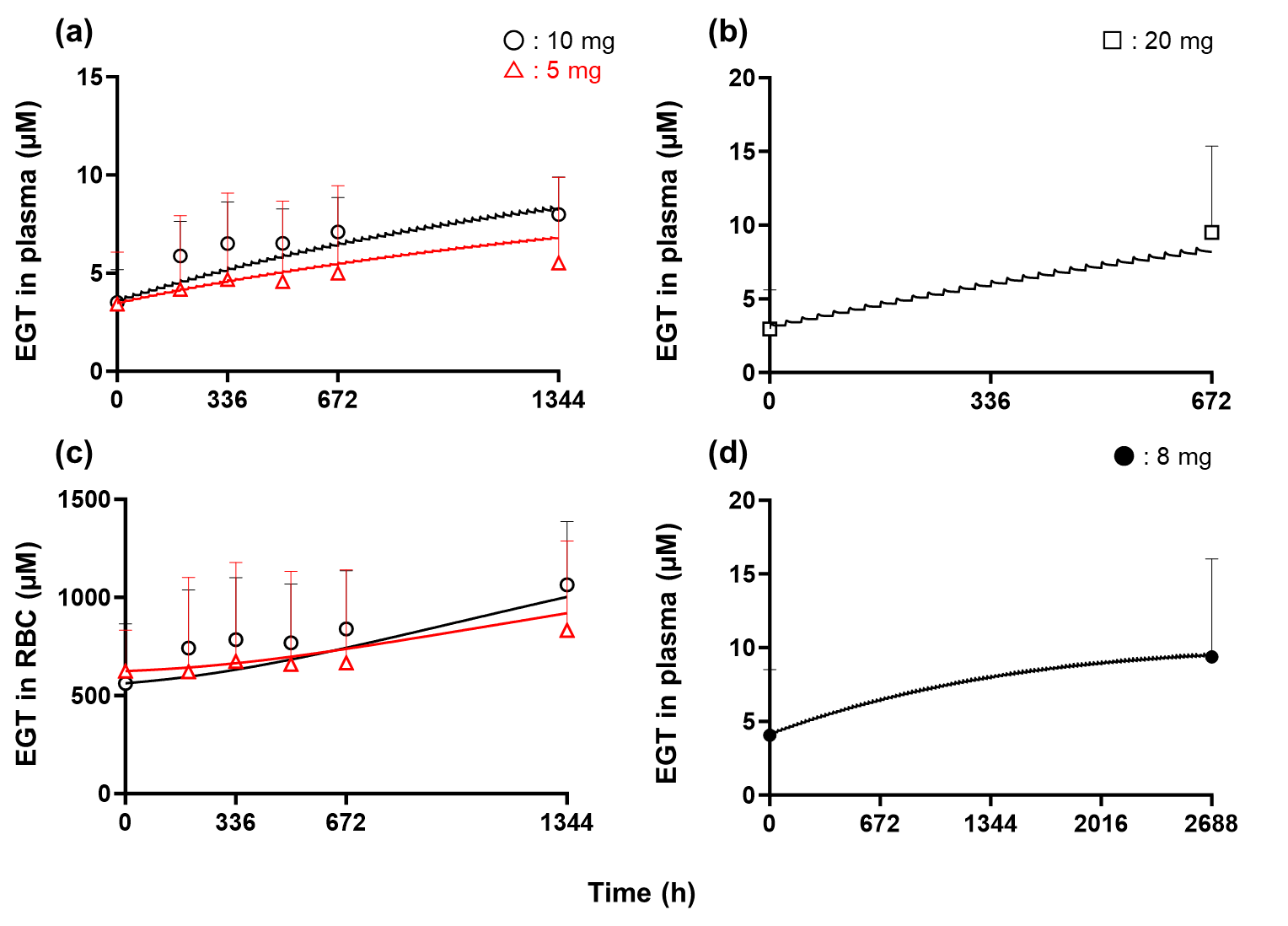


**SUPPLEMENTARY FIGURE 2** Final fitting of the PBPK model to the EGT concentration profiles. (A, C) Open triangles and circles represent EGT concentrations in plasma (A) and RBC (C) obtained in Study 1 (5 and 10 mg/day, respectively). (B) Squares represent plasma concentrations obtained in previous clinical studies (20 mg/day). (D) Closed circles represent plasma concentrations obtained in Study 2 (8 mg/day). The solid lines represent a fitted curve. Each point represents mean ± standard deviation (n=14–50). EGT, ergothioneine; PBPK, physiologically-based pharmacokinetic.

## Supplementary Tables

**SUPPLEMENTARY TABLE 1** Validation of plasma.

| Validation items | Result | | | |
| --- | --- | --- | --- | --- |
| Calibration curve  (weighting; 1/x^2^) | Correlation coefficient (r) | | Accuracy | |
|  | 0.9974 to 0.9983 | | LLOQ: 95.8% to 106.0% | |
|  |  |  | Others: 90.0% to 109.0% | |
| Accuracy and precision  (Intra-assay reproducibility) | Precision (CV) | | Accuracy | |
|  | LLOQ: 4.7% | | LLOQ: 107.0% | |
|  | Others: 1.9% to 4.0% | | Others: 99.5% to 103.0% | |
| Accuracy and precision  (Inter-assay reproducibility) | Precision (CV) | | Accuracy | |
|  | LLOQ: 4.6% | | LLOQ: 109.0% | |
|  | Others: 5.1% to 9.3% | | Others: 106.0% to 107.9% | |
| Material effect | Precision (CV) | | Accuracy | |
|  | 6.9% | | 96.0% | |
| Additive recovery | Accuracy | | | |
|  | 1 μmol/L: 90.2% | | 5 μmol/L: 96.3% | |
| Carry-over | Analyte | | I.S. | |
|  | 1^st^ | 19.2% to 29.7% | 1^st^ | 0.2% to 0.3% |
|  | 2^nd^ | 6.4% to 18.2% | 2^nd^ | 0.1% to 0.2% |
|  | 3^rd^ | 7.3% to 15.3% | 3^rd^ | 0.0% to 0.1% |
| Dilution integrity  (10-fold dilution) | Precision (CV) | | Accuracy | |
|  | 9.7% | | 94.6% | |
| Post-preparative stability in an autosampler  (set temperature; 10 °C) | Period (h) | | Accuracy | |
|  | 48 | | 1 μmol/L: 90.7% | |
|  |  |  | 5 μmol/L: 92.5% | |
| Freeze and thaw stability | Period (cycles) | | Accuracy | |
|  | 3 | | 1 μmol/L: 95.7% | |
|  |  |  | 5 μmol/L: 99.6% | |
| Short-term stability at room temperature  (acceptable range: 1.0 to 30.0 °C) | Period (h) | | Accuracy | |
|  | 24 | | 1 μmol/L: 89.2% | |
|  |  |  | 5 μmol/L: 91.2% | |
| Long-term stability in a deep freezer  (acceptable range: -90.0 to -70.0 °C) | Period (days) | | Accuracy | |
|  | 93 | | 1 μmol/L: 102.8% | |
|  |  |  | 5 μmol/L: 110.6% | |

CV, coefficient of variation; LLOQ, lower limit of quantification.

**SUPPLEMENTARY TABLE 2** Validation items for blood.

| Validation items | Result | | | |
| --- | --- | --- | --- | --- |
| Calibration curve  (weighting; 1/x^2^) | Correlation coefficient (r) | | Accuracy | |
|  | 0.9997 to 0.9999 | | LLOQ: 99.7% to 101.3% | |
|  |  |  | Others: 97.5% to 103.0% | |
| Accuracy and precision  (Intra-assay reproducibility) | Precision (CV) | | Accuracy | |
|  | LLOQ: 3.4% | | LLOQ: 107.0% | |
|  | Others: 0.4% to 1.7% | | Others: 100.3% to 100.7% | |
| Accuracy and precision  (Inter-assay reproducibility) | Precision (CV) | | Accuracy | |
|  | LLOQ: 3.8% | | LLOQ: 104.7% | |
|  | Others: 1.0% to 2.7% | | Others: 99.6% to 100.5% | |
| Material effect | Precision (CV) | | Accuracy | |
|  | 1.5% | | 100.1% | |
| Additive recovery | Accuracy | | | |
|  | 30 μmol/L: 100.0% | | 150 μmol/L: 97.2% | |
| Carry-over | Analyte | | I.S. | |
|  | 1^st^ | 9.4% to 15.5% | 1^st^ | 0.0% |
|  | 2^nd^ | 2.4% to 7.6% | 2^nd^ | 0.0% |
|  | 3^rd^ | 1.9% to 3.3% | 3^rd^ | 0.0% |
| Dilution integrity  (100-fold dilution) | Precision (CV) | | Accuracy | |
|  | 1.6% | | 97.0% | |
| Post-preparative stability in an autosampler  (set temperature; 10 °C) | Period (h) | | Accuracy | |
|  | 48 | | 30 μmol/L: 100.0% | |
|  |  |  | 150 μmol/L: 101.4% | |
| Freeze and thaw stability | Period (cycles) | | Accuracy | |
|  | 3 | | 30 μmol/L: 91.0% | |
|  |  |  | 150 μmol/L: 93.0% | |
| Short-term stability at room temperature  (acceptable range: 1.0 to 30.0 °C) | Period (h) | | Accuracy | |
|  | 24 | | 30 μmol/L: 88.5% | |
|  |  |  | 150 μmol/L: 104.2% | |
| Long-term stability in a deep freezer  (acceptable range: -90.0 to -70.0 °C) | Period (days) | | Accuracy | |
|  | 93 | | 30 μmol/L: 95.9% | |
|  |  |  | 150 μmol/L: 109.9% | |

CV, lower limit of quantification; LLOQ, lower limit of quantification.

**SUPPLEMENTARY TABLE 3** Baseline characteristics of the study participants in Study 1.

|  |  |  | 5 mg (n=14) | | |  | 10 mg (n=14) | | | P-value |
| --- | --- | --- | --- | --- | --- | --- | --- | --- | --- | --- |
| Male/Female |  |  |  | 10 / 4 |  |  |  | 10 / 4 |  | 1.00 |
| Age (years) |  |  | 42.4 | ± | 7.9 |  | 42.1 | ± | 8.1 | 0.91 |
| Height (cm) |  |  | 167.3 | ± | 7.9 |  | 168.7 | ± | 9.4 | 0.67 |
| Weight (kg) |  |  | 61.2 | ± | 10.7 |  | 65.7 | ± | 12.5 | 0.32 |
| BMI (kg/m^2^) |  |  | 21.8 | ± | 3.1 |  | 23.0 | ± | 3.3 | 0.35 |
| Plasma EGT (μM) | |  | 3.42 | ± | 1.7 |  | 3.51 | ± | 2.8 | 0.92 |
| Blood EGT (μM) |  |  | 285.9 | ± | 126.3 |  | 265.9 | ± | 105.0 | 0.65 |

Data are presented as means ± standard deviations. BMI, body mass index; EGT, ergothioneine.

**SUPPLEMENTARY TABLE 4** Change in each item of curiosity.

|  |  | Group | *N* | Baseline | | | Week 16 | | | Δ values | | | P-value |
| --- | --- | --- | --- | --- | --- | --- | --- | --- | --- | --- | --- | --- | --- |
| **JCEI** | |  |  |  |  |  |  |  |  |  |  |  |  |
|  | Stretching | placebo | 49 | 2.23 | ± | 0.11 | 2.27 | ± | 0.12 | 0.04 | ± | 0.10 | 0.685 |
|  |  | EGT | 50 | 2.17 | ± | 0.12 | 2.25 | ± | 0.11 | 0.09 | ± | 0.07 |  |
|  | Embracing | placebo | 49 | 2.27 | ± | 0.10 | 2.38 | ± | 0.12 | 0.11 | ± | 0.10 | 0.587 |
|  |  | EGT | 50 | 2.30 | ± | 0.12 | 2.47 | ± | 0.12* | 0.18 | ± | 0.07 |  |
|  | CEI-II | placebo | 49 | 2.25 | ± | 0.10 | 2.33 | ± | 0.11 | 0.08 | ± | 0.09 | 0.603 |
|  |  | EGT | 50 | 2.24 | ± | 0.11 | 2.37 | ± | 0.11* | 0.14 | ± | 0.07 |  |
| **Epistemic Curiosity Scale** | |  |  |  |  |  |  |  |  |  |  |  |  |
|  | Diverse | placebo | 49 | 3.40 | ± | 0.10 | 3.32 | ± | 0.13 | -0.09 | ± | 0.09 | 0.471 |
|  |  | EGT | 50 | 3.57 | ± | 0.10 | 3.57 | ± | 0.11 | -0.01 | ± | 0.07 |  |
|  | Specific | placebo | 49 | 3.30 | ± | 0.09 | 3.29 | ± | 0.10 | -0.01 | ± | 0.07 | 0.083 |
|  |  | EGT | 50 | 3.21 | ± | 0.09 | 3.39 | ± | 0.09* | 0.18 | ± | 0.08 |  |
| **Interpersonal Curiosity Scale** | |  |  |  |  |  |  |  |  |  |  |  |  |
|  | personal emotions | placebo | 49 | 3.58 | ± | 0.11 | 3.59 | ± | 0.12 | 0.01 | ± | 0.08 | 0.596 |
|  |  | EGT | 50 | 3.71 | ± | 0.11 | 3.78 | ± | 0.14 | 0.08 | ± | 0.09 |  |
|  | Privacy | placebo | 49 | 2.48 | ± | 0.13 | 2.54 | ± | 0.14 | 0.06 | ± | 0.09 | 0.330 |
|  |  | EGT | 50 | 2.13 | ± | 0.11 | 2.30 | ± | 0.12* | 0.17 | ± | 0.08 |  |
|  | Attributes | placebo | 49 | 2.73 | ± | 0.15 | 2.88 | ± | 0.16 | 0.15 | ± | 0.10 | 0.951 |
|  |  | EGT | 50 | 2.72 | ± | 0.15 | 2.88 | ± | 0.17 | 0.16 | ± | 0.13 |  |

Data are presented as means ± standard deviations. The paired *t*-test was performed for intergroup comparisons from baseline. *P<0.05. P-values indicate differences between groups for changes from baseline to Week 16. Two independent sample *t*-tests were performed. CEI-II, Curiosity and Exploration Inventory-II; EGT, ergothioneine; JCEI, Japanese version of Curiosity and Exploration Inventory.

**SUPPLEMENTARY TABLE 5** Initial values and parameters optimized in the final fitting of the PBPK model.^a^

|  | Values ± SD | Units | Notes |
| --- | --- | --- | --- |
| Daily ingestion rate of EGT via diet, R | 1.52 ± 0.31 | µmol/h | Optimized |
| Maximum uptake rate | | | |
| Duct, V_maxD_  RBC precursor, V_maxP_ | 105 ± 14  11.0 ± 0.9 | µmol/h  µmol/h | Optimized  Optimized |
| Initial values for fitting | | | |
| 5 mg/day  C_Plasma_^b^  C_RBC_^b^  10 mg/day  C_Plasma_^b^  C_RBC_^b^  20 mg/day  C_Plasma_^b^  C_RBC_^c^  8 mg/day  C_Plasma_^b^  C_RBC_^c^ | 3.42  624  3.51  562  2.97  594  4.07  594 | µM  µM  µM  µM  µM  µM  µM  µM | Fixed  Fixed  Fixed  Fixed  Fixed  Fixed  Fixed  Fixed |

^a^Each value represents per human value assuming 70 kg body weight. ^b^The blank value obtained in each clinical study. ^c^The average of blank values at 5 and 10 mg/day. EGT, ergothioneine; PBPK, physiologically-based pharmacokinetic; SD, standard deviation.

# Supplementary information


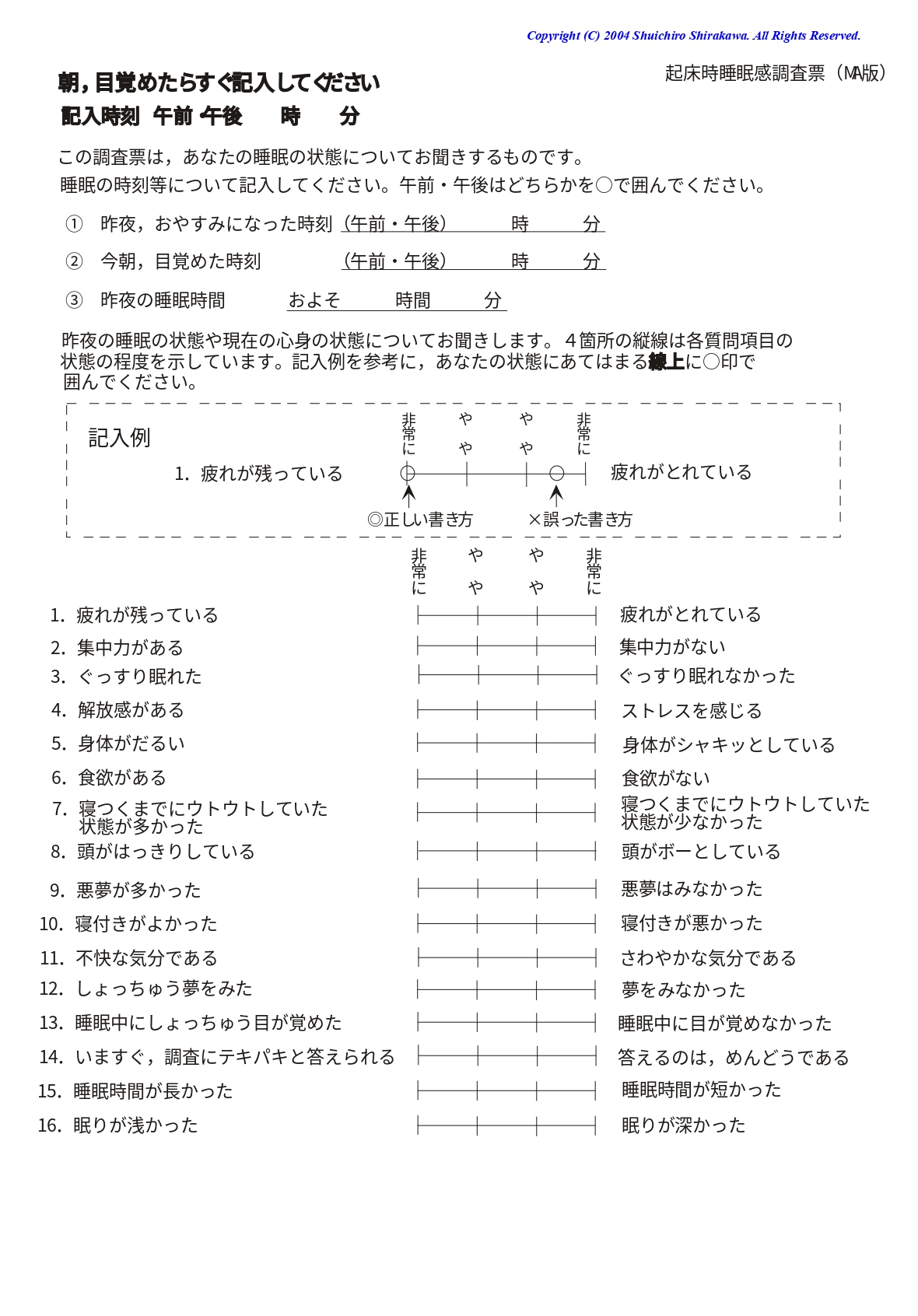

Supplement: Supplementary file 1 — Data S1. [file FSN3-13-e70382-s001.docx]
